# Advances in Cancer Immunotherapy for Solid Tumors

**DOI:** 10.1002/advs.76020

**Published:** 2026-06-09

**Authors:** Shira Gabizon‐Peretz, Harriet M. Kluger

**Affiliations:** ^1^ Medical Oncology Yale University New Haven Connecticut USA; ^2^ Davidoff Cancer Center, Petah Tikva, Faculty of Medicine Tel‐Aviv University Tel Aviv‐Yafo Israel; ^3^ Smilow Cancer Hospital at Yale New Haven New Haven Connecticut USA

**Keywords:** immune checkpoint inhibitors, immunotherapy resistance, solid tumor immunotherapy, perioperative immunotherapy, bispecific antibodies, cellular therapies, cancer vaccines

## Abstract

Cancer immunotherapy has fundamentally transformed the management of solid tumors, ranging from immune checkpoint blockade to a broader spectrum of immune‐modulating strategies. While inhibitors of CTLA‐4 and the PD‐1/PD‐L1 axis remain central to clinical practice, heterogeneous clinical responses, immune‐related toxicities, and different resistance mechanisms underscore the need for next‐generation approaches. This review integrates recent advances in cancer immunotherapy for solid tumors, with an emphasis on emerging biological concepts and therapeutic platforms that extend beyond classical checkpoint inhibition. We discuss novel immune checkpoints, biomarker‐driven approvals, and the expanding role of immunotherapy in different disease settings. Antibody‐based platforms are highlighted as strategies that integrate direct tumor targeting with immune activation, which have reshaped standards of care in several malignancies. We further review advances in adoptive cellular therapies as well as next‐generation cytokine therapies and cancer vaccines aimed at enhancing tumor‐specific immune responses while mitigating systemic toxicity. Finally, we address key unresolved challenges, including mechanisms of resistance, optimization of sequencing and dosing strategies, and clinical trial design considerations. Together, these developments reflect a rapidly evolving field focused on broadening efficacy, improving safety, and personalizing treatment in solid tumors.

## Graphical abstract

Cancer immunotherapy for solid tumors is rapidly evolving beyond classical checkpoint blockade toward more precise strategies. Emerging checkpoint targets, bispecific antibodies, antibody–drug conjugate combinations, personalized vaccines, cellular therapies, and engineered cytokines are expanding therapeutic possibilities, while biomarker‐guided selection and response‐adapted approaches aim to overcome resistance, improve safety, and broaden clinical benefit.

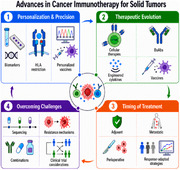

## 1 Introduction

Over the past two decades, cancer immunotherapy has reshaped the therapeutic landscape for solid tumors, transitioning from a niche concept to a mainstay of systemic treatment. Older immune‐stimulatory strategies such as high‐dose interleukin‐2 (IL‐2) in metastatic melanoma and renal cell carcinoma provided the first clinical proof‐of‐concept that durable immune‐mediated tumor regression was possible, albeit in a small subset of patients and at the cost of considerable toxicity [1]. The subsequent advent of immune checkpoint inhibitors (ICIs)—initially targeting cytotoxic T‐lymphocyte–associated antigen 4 (CTLA‐4), and later programmed death 1 (PD‐1) and its ligand PD‐L1—marked a turning point [2, 3, 4, 5]. By releasing inhibitory brakes on T‐cells and restoring effector function within the tumor microenvironment, these agents produced unprecedented improvements in overall survival (OS), with long‐term survivors in diseases such as melanoma, non‐small cell lung cancer (NSCLC) and renal cell carcinoma.

Despite this transformative progress, the clinical impact of checkpoint blockade remains heterogeneous. A substantial proportion of patients either fail to respond to ICI therapy or develop progression after an initial response. Immune‐related adverse events (irAEs)—ranging from mild, organ‐limited toxicities to severe, irreversible endocrinopathies, neurologic events, or life‐threatening pneumonitis and myocarditis—pose additional challenges, particularly when ICIs are deployed in earlier stages of disease in patients who might be cured with surgery or other modalities. Moreover, key patient subgroups, including those with active brain metastases, performance status limitations, or dependency on corticosteroids, remain underrepresented in pivotal trials and may derive less benefit in real‐world practice. Collectively, these limitations have catalyzed intensive efforts to refine patient selection, expand the repertoire of immunotherapeutic targets, and develop more combinations and sequencing strategies.

Beyond the classical CTLA‐4 and PD‐1/PD‐L1 axis, next‐generation immunotherapeutic strategies have been or are being explored to overcome resistance and broaden the spectrum of responsive tumors. These include new checkpoint targets such as LAG‐3, TIM‐3, and TIGIT; structurally optimized PD‐1/PD‐L1 antibodies with modified Fc domains; and combinations of ICIs with targeted agents or antiangiogenic therapies that modulate the tumor microenvironment. In parallel, antibody‐based platforms have expanded far beyond unconjugated monoclonal antibodies to encompass bispecific antibodies and T‐cell engagers, biparatopic constructs, and antibody–drug conjugates (ADCs) that link cytotoxic payloads to tumor‐directed antibodies. Several of these approaches have already led to meaningful improvements in survival and reshaped the standard of care in specific disease settings.

A further frontier is represented by advanced cellular and immune‐activating therapies, including tumor‐infiltrating lymphocyte (TIL) therapy that offers a polyclonal, tumor‐reactive T‐cell product derived directly from the patient's tumor, or chimeric antigen receptor (CAR) T‐cell therapies and T‐cell receptor (TCR)–engineered T‐cells. While CAR and TCR therapies have been most successful to date in hematologic malignancies, they are now being adapted to solid tumors. In addition, there is a renewed interest in cytokine‐based therapies and cancer vaccines, including engineered cytokines and neoantigen‐directed vaccine platforms, which aim to amplify or initiate tumor‐specific immunity in a more controlled and tolerable manner than earlier systemic cytokine approaches.

**FIGURE 1:**
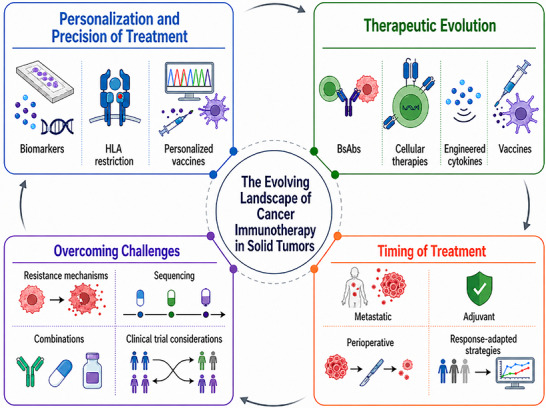
The Evolving Landscape of Cancer Immunotherapy in Solid Tumors. Conceptual overview of the major directions shaping cancer immunotherapy in solid tumors. The figure summarizes four major themes: personalization and precision of treatment through biomarkers, HLA‐restricted approaches, and individualized vaccine strategies; therapeutic evolution through bispecific antibodies, cellular therapies, cytokine‐based approaches, and vaccines; expansion across treatment settings from metastatic to adjuvant, neoadjuvant/perioperative, and response‐adapted strategies; and efforts to overcome resistance, optimize sequencing and combinations, and improve toxicity management.

In this review, we provide an overview of key advances in cancer immunotherapy for solid tumors, with a focus on how these developments are changing clinical practice and future research directions (Figure 1). We begin by summarizing the evolution of immune checkpoint blockade, including the emergence of new inhibitory pathways and refinements in antibody design. We then discuss biomarker‐driven, tissue‐agnostic indications and the ways in which immunotherapy is reshaping treatment paradigms in both metastatic and early‐stage disease. Subsequent sections discuss combinations of immunotherapy with targeted agents and ADCs, as well as the expanding role of adoptive cellular therapy, cytokine‐based strategies, and therapeutic cancer vaccines. Finally, we highlight major challenges and open questions—including mechanisms of primary and secondary resistance, optimal integration of perioperative immunotherapy and trial design considerations—that will be critical in guiding the next studies in this rapidly evolving field.

## 2 Immune Checkpoint Blockade

### 2.1 Approvals of New Targets: LAG‐3

The identification of CTLA‐4 and PD‐1/PD‐L1 as key immune checkpoints revolutionized cancer therapy and established immunotherapy as a new treatment modality. These checkpoints are central regulators of T‐cell activation and peripheral tolerance, and their therapeutic blockade enables the restoration of anti‐tumor immune responses in a subset of. Ipilimumab, the first anti‐CTLA‐4 antibody, demonstrated clinical activity in metastatic melanoma and was approved in 2011 [4], followed by the widespread clinical adoption of anti‐PD‐1 and anti‐PD‐L1 agents across multiple tumor types(2).

**FIGURE 2:**
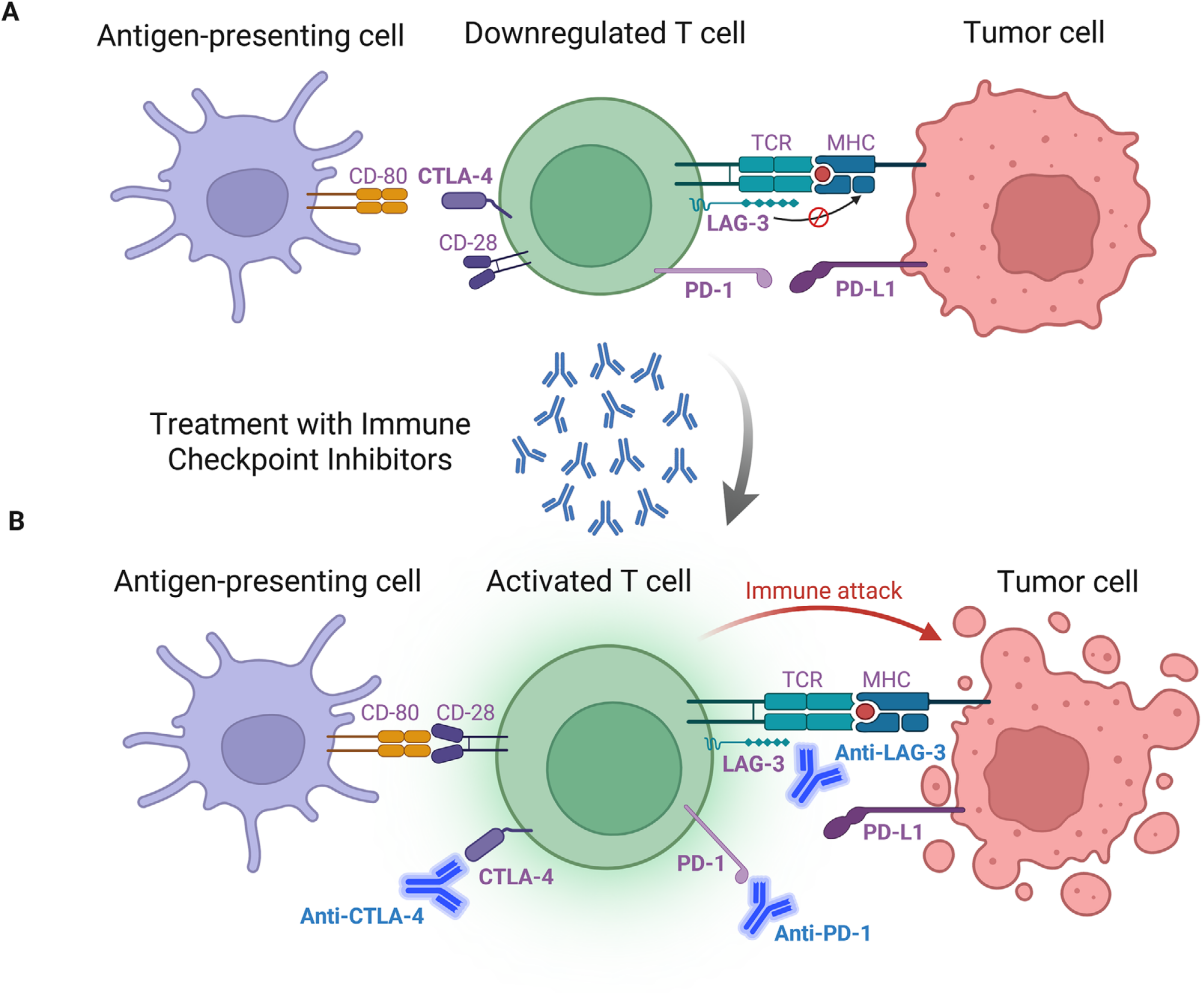
Main Pathways Affected by Approved Immune Checkpoint Inhibitors—PD‐1‐PD‐L1, CTLA‐4, LAG‐3 (A) Inhibitory signaling that limits T‐cell activation in the tumor microenvironment. CTLA‐4 competes with CD28 for CD80 binding on antigen‐presenting cells, while PD‐1/PD‐L1 interactions and LAG‐3 engagement with MHC molecules suppress T‐cell receptor signaling and effector function. (B) Immune reactivation following checkpoint blockade. Inhibition of CTLA‐4 restores costimulatory signaling during T‐cell priming, whereas PD‐1 and LAG‐3 blockade relieves suppressive signaling at the tumor–T‐cell interface, resulting in enhanced T‐cell activation and effective antitumor immune responses.

Despite durable responses in some patients, ongoing research is directed toward identifying additional co‐inhibitory immune checkpoints beyond CTLA‐4 and PD‐1/PD‐L1, with the goal of expanding therapeutic strategies and addressing diverse mechanisms of treatment resistance. Lymphocyte activation gene 3 (LAG‐3) is an inhibitory immune checkpoint receptor expressed on activated T‐cells, regulatory T‐cells, B cells, and natural killer cells. Engagement of LAG‐3 with major histocompatibility complex class II (MHC II) molecules suppresses the activity and proliferation of CD4^+^ effector T‐cells as well as antigen‐specific CD8^+^ T‐cells [6] (Figure 2) [7]. By inhibiting T‐cell receptor (TCR) signaling and cytokine production, including IL‐2 and IFN‐γ, LAG‐3 reduces T‐cell activation and proliferation. Its expression is particularly notable in exhausted T‐cells, where it further contributes to T‐cell suppression [8, 9]. Additional LAG‐3 ligands and signaling interactions, including FGL1, galectin‐3, and effects on immune synapse signaling, further support its role as a regulator of antitumor immunity, although their relative clinical importance remains less clearly defined [10, 11, 12, 13].

Tumor cells often use LAG‐3 to evade immune surveillance, highlighting it as a possible therapeutic target [14]. The primary clinical benefit of LAG3 inhibition has been demonstrated when used in combination with PD‐1 inhibitors and not as a monotherapy. Relatlimab is the first LAG‐3 targeted inhibitor approved by the FDA on March 2022, based on RELATIVITY‐047 trial (NCT03470922) [15], comparing nivolumab monotherapy to the combination of nivolumab/relatlimab in first line advanced or metastatic melanoma. Combination therapy improved median progression‐free survival (mPFS) to 10.1 months vs. 4.6 months with nivolumab alone (HR 0.75; 95% CI 0.62–0.92), and increased objective response rate (ORR) to 43.1% vs. 32.6%. The 4‐year update showed that the OS benefit remained numerically but not statistically significantly higher for the combination (4‐year OS: 52.0% vs. 42.8%; HR 0.80, 95% CI 0.66–0.99) [16].

Grade 3 or 4 treatment‐related adverse events occurred more frequently in the nivolumab/relatlimab group (18.9% vs. 9.7% with nivolumab only), but substantially less frequently than with the combination of anti‐CTLA‐4 and anti‐PD‐1 [2, 17].

Pharmacovigilance analyses also suggest that nivolumab/relatlimab has a distinct toxicity profile compared with ipilimumab/nivolumab, but was associated with uncommon immune‐mediated events, including vascular, cardiac andneuromuscular toxicity [18]. These findings may be influenced by several confounders, such as greater awareness of irAEs and increased use in patients with autoimmunity [19].

Unfortunately, this combination does not appear to be active in primary or secondary resistance to ICIs with an ORR of 12.0% (95% CI, 8.8–15.8) in patients with progression on ICIs [20]. This limited activity may reflect the fact that established PD‐1 resistance is often driven by much broader mechanisms than LAG‐3 co‐expression.

Regarding intracranial activity, the 2‐year subgroup analysis of the RELATIVITY ‐047 trial suggested that nivolumab/relatlimab combination extends the interval before developing central nervous system (CNS) metastasis compared with those treated with nivolumab alone (11.1 vs. 6.6 months). Retrospective real‐world data demonstrated intracranial clinical benefit (including stable disease) of less than 50%, concordant with extracranial response [21]. An ongoing phase II trial (BLUEBONNET, NCT05704647) is assessing the nivolumab/relatlimab combination in patients with active melanoma brain metastases [22].

Treatment with the nivolumab/relatlimab combination is now being investigated in the perioperative setting with promising results (NCT02519322) [23]. In this study, 30 patients with resectable clinical stage III or oligometastatic stage IV melanoma received two doses of neoadjuvant therapy, yielding a 57% pathologic complete response (pCR) rate and a 70% overall pCR, with no grade 3–4 immune‐related adverse events observed during the neoadjuvant phase. At four years, 80% of patients remained event‐free, including 95% of patients who achieved a major pathological response [24]. This suggests that LAG‐3 blockade may have a role in earlier settings, although larger randomized studies are needed. This combination was also tested in the NEOpredict‐Lung (NCT04205552) randomized phase 2 trial with signals of clinical and biological activity, and the authors concluded that further exploration of dual targeting of PD‐1 and LAG‐3 in NSCLC is clearly warranted [25].

Other than advanced melanoma, there are no additional FDA‐approved indications for relatlimab at present. Additional anti–LAG‐3 antibodies are also being evaluated, including fianlimab [26, 27]. Several alternative approaches to LAG‐3 targeting are under investigation. Eftilagimod alpha, a soluble LAG‐3–Ig fusion protein, differs mechanistically by activating antigen‐presenting cells through MHC class II engagement rather than blocking inhibitory LAG‐3 signaling on T‐cells; in HNSCC, its combination with pembrolizumab in the TACTI‐003 study has shown a favorable safety profile and early efficacy signals, including in patients with low PD‐L1 expression [28, 29],

Tebotelimab, a bispecific antibody targeting both PD‐1 and LAG‐3, represents another conceptually interesting strategy, as it may clarify whether dual checkpoint blockade within a single molecule can improve antitumor activity or alter the therapeutic window compared with separate antibody combinations [30].

In summary, relatlimab has positioned LAG‐3 inhibition as a meaningful addition to modern immunotherapy, offering improved efficacy over PD‐1 monotherapy with a more favorable safety profile than CTLA‐4–based combinations. While its impact on overcoming resistance to PD‐1/PD‐L1 inhibitors is limited, primary evidence for intracranial activity, and encouraging neoadjuvant results highlight its clinical value as an alternative to anti‐CTLA‐4‐based combinations. Exploring additional ways to target the LAG‐3 pathway—including bispecific antibodies, other dual‐checkpoint constructs, and agonistic agents such as eftilagimod alpha – may ultimately yield more potent immune activation in patients previously exposed to anti‐PD‐1 therapy.

### 2.2 Tissue‐Agnostic Approvals of Immune Checkpoint Inhibitors (dMMR/TMB‐H)

In contrast to standard treatment approvals based on the histologic origin of a tumor, tissue‐agnostic approvals have emerged over the past few years. This approach offers several advantages: it represents a form of “precision oncology,” directing treatment toward the specific characteristics of a patient's tumor rather than its histology, and it enables faster expansion of treatment options for rare cancers. Two specific biomarkers—tumor mutational burden and microsatellite instability—have been established as predictive of response to ICIs across multiple solid tumor types, leading to FDA approvals that are independent of tissue of origin [31].

Microsatellite instability (MSI) reflects hypermutability of short, repetitive DNA sequences (microsatellites) resulting from defects in the DNA mismatch repair (MMR) system. MMR genes are responsible for correcting the ubiquitous nucleotide base mispairings and small insertions or deletions that occur during DNA replication. Several MMR genes have been identified, including hMSH2, hMLH1, hPMS1, hPMS2, MSH6, and hMLH3, an MMR gene that interacts with MLH1. High levels of MSI (MSI‐H) or deficient MMR (dMMR) may arise from germline mutations, as seen in Lynch syndrome, leading to a lifelong systemic predisposition to MSI‐H tumors across multiple organs. Alternatively, sporadic cases often result from acquired somatic hypermethylation of the MLH1 promoter, causing epigenetic silencing [32].

Tumors with MSI‐H or dMMR accumulate insertion/deletion mutations at microsatellite loci, leading to genetic instability and generation of neoantigens. MSI‐H/dMMR tumors are characterized by a high mutational burden and increased neoantigen load, which correlates with sensitivity to ICIs [33]. Clinical data have demonstrated that dMMR/MSI‐H status is associated with high ORR and durable benefit from PD‐1 blockade across multiple tumor types, independent of tissue origin [34]. MSI is detected by PCR, immunohistochemistry, or NGS‐based assays.

**FIGURE 3:**
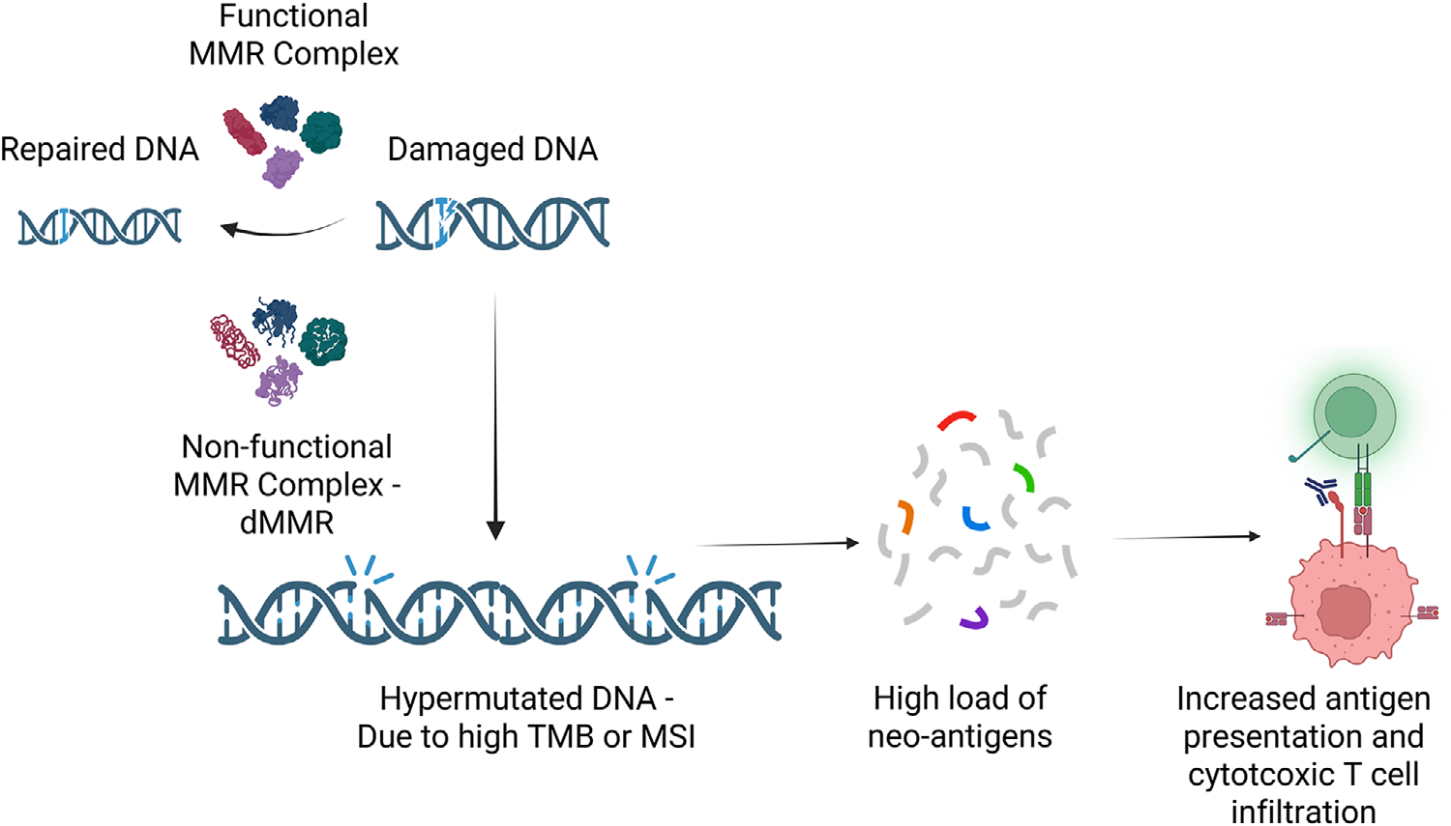
Tumor mutation burden (TMB) and high microsatellite instability (MSI‐H) increase antigen presentation. Functional DNA mismatch repair (MMR) complexes maintain genomic stability by correcting replication errors. In contrast, deficiency of the MMR system (dMMR) results in accumulation of DNA damage, leading to a hypermutated DNA phenotype. This increased mutational load generates a high number of neoantigens, enhancing tumor antigen presentation and promoting cytotoxic T‐cell infiltration and antitumor immune responses.

In contrast to dMMR, which represents a more clearly defined predictive biomarker for immunotherapy response, tumor mutational burden (TMB) reflects a broader and more heterogeneous aspect of tumor biology, defined as the number of somatic mutations per megabase within the cancer genome. The underlying rationale is that higher mutational load increases the likelihood of generating immunogenic neoantigens and effective T‐cell recognition, thereby increasing the likelihood of benefit from immune checkpoint inhibition (Figure 3). This biological rationale is supported by clinical observations in NSCLC and melanoma, including CheckMate 568, where response rates increased with rising TMB irrespective of PD‐L1 expression, and exploratory analyses linking TMB with inflammatory gene signatures and survival [35, 36]; This data fueled early enthusiasm for TMB as a potential tissue‐agnostic biomarker; however, this association has proven insufficient for reliable patient selection. Significant technical and biological limitations—including variability across sequencing platforms, tumor heterogeneity, poor concordance between tissue and blood‐based assays, and the assumption that all mutations are equally immunogenic—have limited its clinical applicability, and real‐world data have not consistently validated TMB as a standalone predictor of immunotherapy benefit [37, 38, 39, 40, 41, 42].

Further highlighting the spectrum of immunogenic tumor biology, a more specific ultramutated subgroup worth noting is POLE‐mutated tumors, which are defined by pathogenic alterations in the exonuclease (proofreading) domain of the POLE gene, most commonly at codons 286, 411, 297, 456, and 459 (“hotspot” mutations) [43]. These tumors occur most frequently in endometrial cancer (up to 10%) but are also observed in colorectal and other solid tumors at lower frequencies. POLE‐mutant tumors exhibit an ultramutated phenotype with extremely high tumor mutational burden and dense T‐lymphocyte infiltration, reflecting a profoundly immunogenic microenvironment. This biological profile likely underlies their excellent prognosis, despite frequent association with high‐grade histology and other adverse clinicopathologic features [44]. Importantly, clinical observations suggest that these patients derive minimal benefit from chemotherapy—particularly in the adjuvant setting—while exhibiting exceptionally high response rates to immunotherapy [45].

KEYNOTE‐158 was an open‐label, multicohort phase 2 trial evaluating pembrolizumab monotherapy in previously treated advanced solid tumors, prospectively assessing TMB and MSI‐H/dMMR status as predictive biomarkers of response [46, 47]. TMB‐high tumors were most frequently observed in small cell lung carcinoma (SCLC), cervical, and anal cancers. Pembrolizumab showed higher response rates in TMB‐high versus non–TMB‐high tumors (ORR 29% vs. 6%), as well as durable clinical benefit in MSI‐H/dMMR disease with prolonged responses and survival (mean duration of response >47 months; mOS >20 months). A plateau of the latter portion of both the PFS and OS curves in the TMB‐high group may indicate that a portion of responders after exposure to immunotherapy maintain durable long‐term disease control [48]. Key limitations include the single‐arm design without a comparator, the inclusion of numerous tumor types with small cohort sizes, and the underrepresentation of more common malignancies.

Given that KEYNOTE‐158 largely excluded dMMR colorectal cancer, subsequent trials—most notably KEYNOTE‐177 and CheckMate‐8HW—provided the critical evidence needed to define the role of immunotherapy in this population. KEYNOTE—177 was a phase 3 study that compared single‐agent PD‐1 blockade with Pembrolizumab with chemotherapy as first‐line therapy for MSI‐H–dMMR advanced colorectal cancer. Pembrolizumab treatment resulted in a major improvement in mPFS [49], which subsequently translated into improved survival, with a mOS was 77.5 months with pembrolizumab versus 36.7 months with chemotherapy (HR, 0.73; 95% CI 0.53‐0.99), and a 5‐year OS rate of 54.8% vs. 44.2% [50]. It should be mentioned that crossover after progression was protocol‐defined, but the crossover rate concluded only in 62%. Treatment with immunotherapy, compared to chemotherapy, had a significantly better toxicity profile [49].

The CheckMate‐8HW trial [51] is a phase 3 randomized study evaluating nivolumab/ipilimumab (3mg/1mg dosing) versus nivolumab monotherapy and chemotherapy in metastatic colorectal cancer with MSI‐H∖dMMR across all lines of treatment. Dual checkpoint blockade significantly improves PFS and ORR compared to chemotherapy and nivolumab alone, with 2‐year PFS of 72% for the combination versus 14% for chemotherapy.

While no direct comparison can be made between CheckMate‐8HW and KEYNOTE‐177 in the absence of a head‐to‐head trial, the inclusion of a single‐agent nivolumab arm in CheckMate‐8HW provides clinically relevant context. Given the observed superiority of the dual checkpoint regimen over monotherapy in this study, it is reasonable to consider nivolumab/ipilimumab as a therapeutic option to reduce the frequency of primary progression, especially when appropriately selected patients with worse prognosis, such as KRAS/NRAS mutant disease or liver metastases [51].

Dostarlimab, another anti‐PD‐1 monoclonal antibody, has also been demonstrated in this patient population in the GARNET study [52], and more specifically in advanced endometrial cancer in combination with chemotherapy in the RUBY trial [53]. Initial results showed a marked PFS benefit in the dMMR/MSI‐H subgroup (24‐month PFS 61.4% vs. 15.7% with placebo), and subsequent data confirmed an OS benefit in both the dMMR/MSI‐H subgroup and, notably, even in the pMMR population.

In 2022, the primary results of the AZUR‐1 trial led by Cercek were published. This was a phase 2 study of neoadjuvant treatment with dostarlimab monotherapy for locally advanced (Stage 2–3) dMMR rectal adenocarcinoma. All patients (41 reported to date) achieved a clinical complete response, and all proceeded with the “watch‐and‐wait” approach (avoiding subsequent therapy, namely chemoradiotherapy and surgery) [54]. The first twenty patients recruited achieved a sustained clinical complete response with a median follow‐up of 28.9 months (95% CI 22.9–37.1) from their first treatment. AZUR‐2 is a randomized phase III trial evaluating the efficacy of perioperative dostarlimab compared with standard of care in participants with untreated T4N0 or Stage III dMMR/MSI‐H colon cancer [55]. Cercek has also initiated a study of neo‐adjuvant and non‐operative management of all early‐stage dMMR solid tumors, regardless of tumor site, with promising results [56].

Although non‐operative management is currently established mainly in rectal cancer, discussions regarding surgical de‐escalation post neo‐adjuvant treatment are emerging across other malignancies, such as melanoma, HNSCC, and lung [57, 58, 59]. In highly immunogenic tumors that demonstrate high clinical response rates to immunotherapy, it is plausible that watch‐and‐wait strategies may eventually become relevant in the future, pending supportive prospective data. These evolving considerations across multiple tumor types reflect a broader movement toward response‐adapted, organ‐preserving strategies in the immunotherapy era.

### 2.3 Integration of Immune Checkpoint Inhibitors with Chemotherapy and Targeted Therapies Across Tumor Types

The incorporation of ICIs to established systemic therapies has become an important strategy across several solid tumors, based on the complementary effects of cytotoxic or targeted agents and immune checkpoint blockade. Chemotherapy can promote antigen release and immune priming, while targeted therapies may modulate oncogenic signaling and angiogenesis, thereby increasing sensitivity to ICIs.

The management of unresectable hepatocellular carcinoma (HCC) has transitioned from single‐agent tyrosine kinase inhibitors (TKIs) to immune checkpoint inhibitor (ICI)–based combination regimens, leading to improved outcomes. Historically, sorafenib served as the only approved systemic therapy and provided a limited survival benefit. More recent pivotal trials, including IMbrave150 [60], showed that the combination of atezolizumab (anti‐PD‐L1) and bevacizumab (anti‐VEGF), and the HIMALAYA [61, 62] study (single‐dose tremelimumab, anti‐CTLA‐4 followed by durvalumab maintenance), significantly improved PFS and OS compared with sorafenib. Median OS in metastatic HCC has increased modestly but meaningfully, reaching 16.4–19.2 months with atezolizumab plus bevacizumab versus 13.4–13.8 months with sorafenib. Durvalumab plus tremelimumab is preferred for patients at high risk of bleeding or those with contraindications to VEGF inhibition.

In SCLC, the incorporation of immunotherapy represents the first major advance in patient outcomes in decades. For many years, platinum‐based chemotherapy with etoposide remained the standard treatment for extensive‐stage disease, but it provided only short‐lived responses and rapid relapse, with poor long‐term survival of approximately 8–10 months [63]. The addition of PD‐L1 inhibitors such as atezolizumab and durvalumab to platinum‐etoposide has now become the standard of care for extensive‐stage SCLC, supported by randomized phase III trials such as IMpower133 [64] and CASPIAN [65], which demonstrated a statistically significant improvement in OS and more durable responses compared with chemotherapy alone (Table 1). Further improvements in outcomes for SCLC with bispecific T‐cell engagers will be discussed later in this review.

**TABLE 1:** Examples of disease settings in which immunotherapy containing regimens have improved survival in recent years.

Standard of care before immunotherapy	Median overall survival (response rate)	Type of cancer	New median overall survival (response rate)	Standard of care including immunotherapy
Platinum based chemotherapy	16 m (50%)	**Metastatic Urothelial Carcinoma**	31.5 m (68%)	Enfortumab vedotin + Pembrolizumab
5FU based chemotherapy	36.7 m (33%)	**Metastatic MSI‐H Colorectal Carcinoma**	77.5 m (45%–68%)	Pembrilizumab Ipilimumab and Nivolumab
Platinum based chemotherapy	9–10 m (70%)	**Extensive Small Cell Lung Cancer**	25.3 m (70%)	Platinum Based Chemotherapy + Atezolizumab or Durvalumab + Tarlatamab maintenance
Sorafenib or Lenvatinib	12 m (10%)	**Metastatic Hepatocellular Carcinoma**	16–24 m (20%–36%)	Atezolizumab + Bevacizumab Durvalumab and Tremelimumab or Ipilimumab and Nivolumab

5FU = 5‐fluorouracil, MSI = Microsatellite Instability

Beyond HCC and SCLC, chemo‐immunotherapy has become a major treatment backbone in several common solid tumors such as NSCLC, TNBC, gastric cancer, and others. These examples are discussed in greater detail in later sections of this review, and they further illustrate the broad clinical impact of adding checkpoint blockade to established systemic therapies.

Attempts to combine immunotherapy with small‐molecule inhibitors have led to several approved regimens in advanced solid tumors. The rationale is to leverage the rapid, high response rates of targeted agents together with the durable responses of immunotherapy, aiming for synergistic efficacy and overcoming resistance mechanisms. Many regimens combining VEGF‐R inhibitors in renal cell carcinoma with PD‐1 inhibitors have been approved. Response rates are higher, but to date, it is unclear whether long‐term survival is superior to ipilimumab and nivolumab (Table 2). These combinations are often used in the frontline setting in patients needing immediate tumor burden reduction [66, 67, 68].

**TABLE 2:** FDA – Approved immune checkpoint inhibitors and tyrosine kinase inhibitors combinations.

Combination	FDA indication	Pivotal study	Date of approval	Efficacy highlights
**Pembrolizumab + Axitinib**	1^st^ line advanced / metastatic renal cell carcinoma	**KEYNOTE‐426** NCT02853331	04/2019	mPFS 15.7 vs. 11.1 mo (HR 0.69 [95% CI: 0.57–0.84] *p* < 0.0001); 5‐y analysis ‐ mOS 47.2% vs. 40.8% (HR 0.84 [95% CI: 0.71‐0.99] *p* < 0.0001) (vs sunitinib)
**Avelumab + Axitinib**	1^st^ line advanced / metastatic renal cell carcinoma	**JAVELIN Renal 101** NCT02684006	05/2019	mPFS 13.9 vs. 8.5 mo (HR 0.66 [95% CI 0.566–0.769] one‐sided *p* < 0.0001) in PD‐L1+; (vs sunitinib), mOS 44.8 vs. 38.9 mo (HR 0.88 [95% CI 0.643–0.969] one‐sided *P* = 0.0116)
**Nivolumab + Cabozantinib**	1^st^ line advanced / metastatic renal cell carcinoma	**CheckMate 9ER** NCT03141177	01/2021	mPFS 16.4 vs. 8.3 mo (HR 0.58 [95% CI, 0.49–0.70] *p* < 0.001); mOS 46.5 vs. 35.5 mo (HR 0.79 [95% CI, 0.63–0.95] *p* = 0.0196) (vs sunitinib)
**Pembrolizumab + Lenvatinib**	1^st^ line advanced / metastatic renal cell carcinoma	**CLEAR / KEYNOTE‐581** NCT02811861	08/2021	mPFS 23.9 vs. 9.2 mo (HR 0.39 [95% CI: 0.32–0.49] *p* < 0.0001); mOS 53.7 vs. 54.3 but HR 0.79 [95% CI: 0.63–0.99] *p* < 0.042; (CR 16%) (vs sunitinib)
**Pembrolizumab + Lenvatinib**	Advanced endometrial carcinoma (pMMR / not MSI‐H) after prior systemic therapy	**KEYNOTE‐775** NCT03517449	09/2019 accelerated 07/2021 final	mPFS 6.7 vs. 3.8 mo (HR 0.60 [95% CI 0.50–0.72] *p* < 0.0001); OS 18 vs. 12.2 mo (HR 0.7 [95% CI 0.56–0.84] *p* = 0.0001); (vs chemotherapy)

PFS = Progression free survival, mo = Months, HR = Hazard ratio, mOS = Median overall survival, pMMR‐ Proficient mismatch repair, MSI = Microsatellite instability, CR = Complete response

Attempts to combine immune checkpoint inhibitors with BRAF and MEK inhibitors in melanoma have been challenging. Overall, outcomes are better with immunotherapy [69], but declining performance status may require treatment with a rapid response to reduce the burden of disease. Unfortunately, although pre‐clinical data were promising [70], most of the efforts to test these combinations have not succeeded due to higher rates of toxicity. Early attempts to combine ipilimumab with BRAF/MEK inhibitors were discontinued due to excessive hepatotoxicity [71, 72]. Later, several studies tested the triplet of anti‐PD‐1/L1 with BRAF and MEK inhibitors, only with positive results, the IMspire150 [73, 74] trial, which demonstrated a significant improvement in PFS with the addition of atezolizumab to vemurafenib and cobimetinib, but no improvement in OS. G3‐4 AEs rates were high in both the intervention and the control group, but included mainly laboratory abnormalities and overall had a manageable safety profile. The limited clinical success of these triplets likely reflects overlapping toxicities, suggesting that improved sequencing or intermittent targeted therapy strategies may be required.

### 2.4 Changing the Paradigm of Treatment—Introduction of Immunotherapy in Earlier Course of the Disease

In the past decade, the treatment paradigm for most cancer types has changed, with an increasing number of therapies first tested in the metastatic setting being moved to earlier stages of disease management in the peri‐operative setting. This shift has been driven by several key developments.

The success of immunotherapy, targeted therapies, and antibody–drug conjugates in the metastatic setting provided compelling evidence that these agents could induce deep and durable responses, prompting investigation into whether earlier intervention could prevent relapse, eradicate micro‐metastatic disease, and improve cure rates [75].

In NSCLC, use of ICIs have evolved from salvage therapy for metastatic cancers to adjuvant therapy after surgery and to the neoadjuvant setting. For advanced non‐driver mutated NSCLC, first‐line systemic treatment generally consists of immunotherapy, cytotoxic chemotherapy, or a chemo‐immunotherapy combination depending on PD‐L1 expression and histology (squamous versus non‐squamous).

Patients with tumor cell PD‐L1 expression over 50% are eligible for immunotherapy‐only treatment if not severely symptomatic. To date, three ICIs have been approved by the United States (U.S.) FDA as single agents for the treatment of advanced NSCLC: two anti‐PD1 antibodies, pembrolizumab based on the KEYNOTE‐024 trial [76], cemiplimab based on the EMPOWER‐Lung 1 trial [77], and the anti‐PD‐L1 antibody atezolizumab based on the IMpower 110 trial [78]. Dual immunotherapy (nivolumab plus ipilimumab) is FDA‐approved for metastatic NSCLC with PD‐L1 ≥1% [79], although it is not specifically preferred over monotherapy for PD‐L1 ≥50%. Further studies are warranted to optimize treatment selection between monotherapy and dual ICI in untreated metastatic NSCLC, although some evidence suggests that dual therapy increases time to progression in patients with bone metastases and squamous histology [80].

The first integration of immunotherapy in the local setting for lung cancer was for unresectable stage III NSCLC and created a major change in the standard of care. Mostly treated with definitive chemoradiation with high recurrence rates, consolidation therapy with durvalumab tested in the PACIFIC trial significantly improved the 5‐year OS to 42.9% versus 33.4% in the placebo group (HR = 0.72, (95% CI, 0.59–0.89)) [81, 82].

In the setting of a resectable disease, the establishment of adjuvant platinum‐based chemotherapy as the standard of care for stages IB‐III NSCLC was largely based on the LACE meta‐analysis, which demonstrated an absolute survival benefit of 5.4% at 5 years [83]. The IMpower010 is a phase 3 randomized trial comparing one year of atezolizumab with observation after cisplatin‐based chemotherapy in patients with resected stage IB–IIIA NSCLC, irrespective of EGFR/ALK status or PD‐L1 expression [84]. The trial met its primary endpoint, disease‐free survival (DFS), improving 3‐year DFS from 48% to 60% in the PD‐L1 >1% stage II–IIIA group (HR 0.66). This led to FDA approval of adjuvant atezolizumab for stage IB–IIIA NSCLC, with a post‐hoc analysis showing the greatest benefit in the PD‐L1 ≥50% subgroup (HR 0.43) [84].

Immunotherapy was later integrated in the neo‐adjuvant phase, as was evaluated in CheckMate 816 phase 3 randomized study. Patients were randomized to three cycles of Nivolumab plus platinum‐based chemotherapy versus chemotherapy alone, with no adjuvant systemic treatment. The key efficacy outcomes were major and statistically significant change in the pCR rates – 24.0% (95% CI, 18.0–31.0) in the nivolumab plus chemotherapy arm versus 2.2% (95% CI, 0.6–5.6) in the chemotherapy alone arm [59], with an OR of 13.94; 99% CI, 3.49–55.75; *P*<0.001. Five‐year OS was 65.4% with nivolumab plus chemotherapy and 55.0% with chemotherapy alone (HR for death, 0.72; 95% CI, 0.523–0.998; *P* = 0.048) [85]. The study revealed consistent benefit across subgroups and no increase in surgical complications or high‐grade toxicity. As mentioned above, NEOpredict‐Lung is also testing the efficacy of neoadjuvant immunotherapy, comparing nivolumab monotherapy versus nivolumab and relatlimab. Currently, several key studies established perioperative treatment with neoadjuvant chemo‐immunotherapy and adjuvant immunotherapy as the standard of care for resectable disease [86, 87, 88].

**FIGURE 4:**
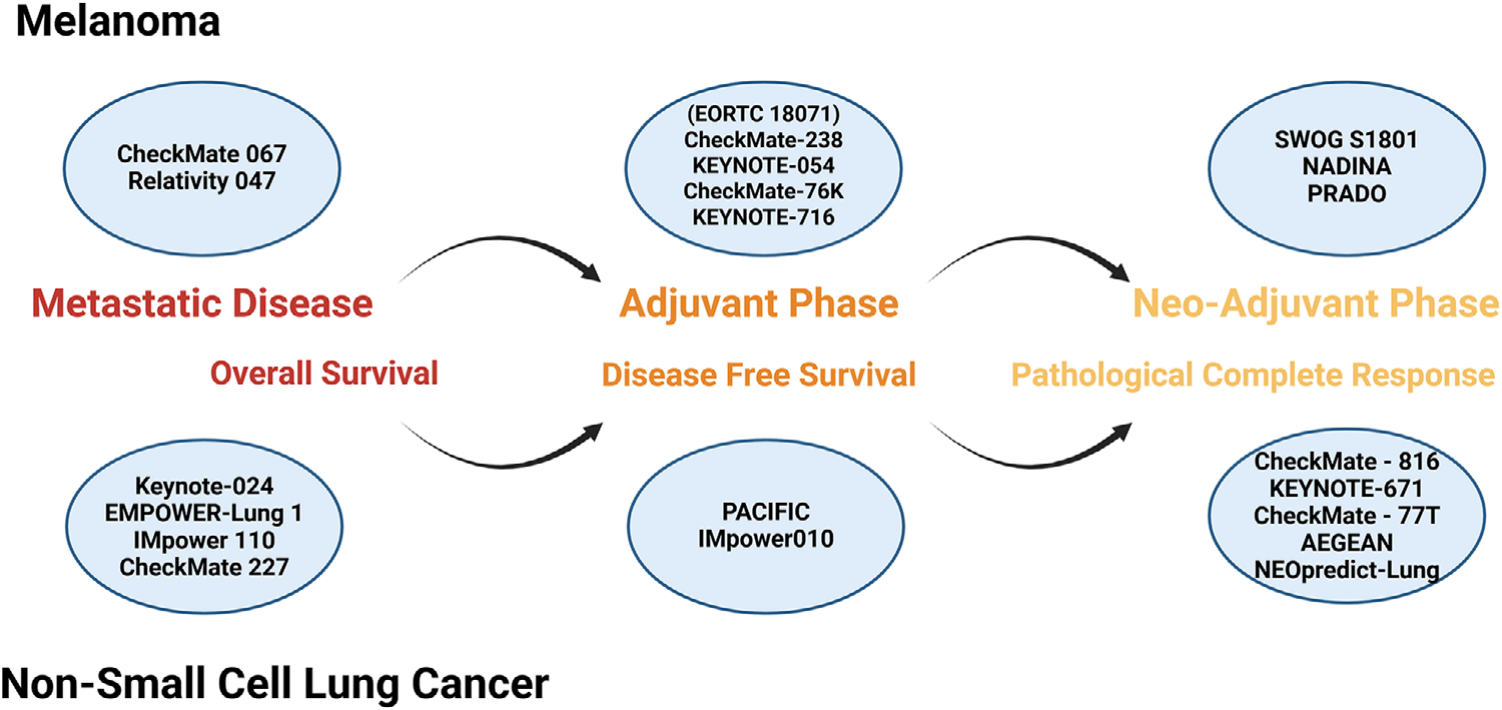
Examples of integration of immunotherapy earlier in the disease course. Representative clinical trials demonstrate the expansion of immune checkpoint inhibitors from the metastatic setting to adjuvant and neoadjuvant treatment across melanoma and non–small cell lung cancer, with corresponding shifts in primary clinical endpoints.

Another example of this paradigm shift is seen in melanoma (Figure 4). Adjuvant anti‐CTLA‐4 or anti‐PD‐1 are superior to observation or interferon gamma in patients with locally advanced disease. Depending on the molecular profile of the tumor, patients can receive targeted treatment (if BRAF is mutated) or immunotherapy. For patients with stage III disease, including microscopic nodal disease, immunotherapy options include nivolumab, based on CHECKMATE‐238 [89, 90] or pembrolizumab, based on KEYNOTE‐054 [91]. Interestingly, adjuvant anti‐PD‐1 has not been shown to improve OS in melanoma, likely due to the ability to effectively eliminate stage IV disease with immune checkpoint inhibitors in patients who did not receive adjuvant anti‐PD‐1, as evidenced by SWOG S1404(93).

Studies have also demonstrated the benefit of adjuvant anti‐PD‐1 for intermediate‐risk disease—stage IIB‐C, in terms of recurrence free survival (RFS) [93, 94, 95]. The follow‐up in these studies remains relatively short, with a 4‐year analysis of pembrolizumab treatment, and reports only RFS. Treatment with single‐agent immunotherapy is tolerated relatively well, but the risk, even if minimal, of irreversible side effects should be weighed heavily against the potential lack of OS benefit, especially in the adjuvant setting.

Some have postulated that neoadjuvant immunotherapy is supported by the concept that initiating ICI while the primary tumor and draining lymph node are still present allows earlier priming of tumor‐specific T‐cells and presentation of a wider range of antigens, which may lead to a stronger and more sustained immune response [96]. Current data suggest that neoadjuvant immunotherapy in melanoma results in high rates of pathological complete response (pCR) and improves event‐free survival compared to adjuvant treatment. However, it is unclear whether the benefit is due to the presence of greater tumor burden at initiation of immunotherapy or whether it is due to earlier initiation of systemic therapy. Two randomized studies have been completed – SWOG S1801 [97] and NADINA [98], both testing perioperative immunotherapy treatment with pembrolizumab or ipilimumab‐nivolumab, respectively. Treatment with dual immunotherapy resulted in an impressive rate of pCR—close to 60%, but with higher rates of G3 and above AEs (almost 30%). Notably, most patients in both studies who did not complete surgical treatment had disease progression, mainly in SWOG S1801, reflecting the possibility of primary resistance to anti PD‐1 treatment.

Both NADINA and the PRADO [57] studies tested the possibility of a response‐mediated approach to tailoring the aggressiveness of treatment—both local (determining the necessity of total lymph node dissection versus index node resection only) and systemic adjuvant treatment, based on the radiological and pathological response of the patient to the neoadjuvant treatment. This method could help reduce overtreatment and potentially reduce secondary resistance rates, which are not yet known in the long‐term metastatic setting after exposure to immunotherapy in the local setting.

### 2.5 Newer Immune Checkpoint Inhibitors

In an attempt to overcome resistance to standard inhibitors of PD‐1/L1, numerous ongoing efforts are geared toward engineering PD‐1 inhibitors to enhance tumor‐specific activity and efforts to target alternative immune checkpoints.

First, there have been several developments in the field of the PD‐1/PD‐L1 pathway involving inhibitors with optimized structures and functionality. Earlier‐generation PD‐1 inhibitors were primarily developed on an IgG4 backbone, which is chosen to minimize Fc‐mediated effector functions but can be less stable and may still retain some residual Fcγ receptor binding. In contrast, newer agents have been specifically engineered to eliminate Fc‐mediated functions altogether. Penpulimab is a humanized anti‐PD‐1 monoclonal antibody engineered on an IgG1 backbone with an inactivated Fc (null) component that eliminates Fcγ receptor binding and Fc‐mediated effector functions, such as antibody‐dependent cellular cytotoxicity (ADCC), Antibody Dependent Cellular Phagocytosis (ADCP), and cytokine release, preventing potential T‐cell elimination. This Fc‐null design aims to improve molecular stability, reduce immunogenicity, and lower the incidence of immune‐related adverse events compared to conventional IgG4‐based PD‐1 inhibitors [99]. It was approved this year for first‐line metastatic nasopharyngeal carcinoma in combination with a platinum agent.

Cosibelimab [100] represents the complete opposite design approach. It is a high‐affinity anti‐PD‐L1 antibody with an unmodified IgG1 Fc domain, enabling both checkpoint blockade and induction of ADCC and complement‐dependent cytotoxicity (CDC) within tumor cells. Even though cosibelimab can theoretically damage effective immune cells [101], clinical safety data have not shown a higher rate of severe immune‐related adverse events compared to other agents, and it has demonstrated high efficacy in the treatment of metastatic squamous cell carcinoma [101], a tumor with relatively high expression of PD‐L1, for which it has received FDA approval. For an overview of immune checkpoint inhibitor–containing regimens approved within the past five years, refer to Table 3.

**TABLE 3:** FDA – Immune checkpoint inhibitors containing regimens approved in the past five years.

Drug / Combination	FDA indication	Pivotal study	Date of approval	Efficacy highlights
Anti – PD‐1				
Pembrolizumab				
Pembrolizumab (adjuvant)	Adjuvant treatment of Stage IIB/IIC **melanoma**	KEYNOTE‐716 NCT03553836	12/2021	36‐mo RFS 76.2% vs. 63.4% (HR 0.62 [95% CI, 0.49‐0.79] *p* = 0.0066); 36‐mo DMFS 84.4% vs. 74.7% (HR = 0.59 [95% CI, 0.44‐0.79] *p* = 0.0029), mRFS and mDMFS not reached (vs placebo)
Pembrolizumab (single agent)	Advanced dMMR/MSI‐H **endometrial cancer** after prior therapy	KEYNOTE‐158 (cohort d) NCT02628067	03/2022	mPFS 13.1 mo [95% CI, 4.3‐34.4], mDOR not reached, ORR 48% [95% CI, 37–60]
Pembrolizumab + platinum‐based chemotherapy → pembrolizumab	Peri‐operative treatment of resectable **NSCLC**	KEYNOTE‐671 NCT03425643	10/2023	mEFS 47.2 vs. 18.3 mo (HR 0.59 [95% CI 0.48‐0.72]), 36‐mo OS 71% vs. 64%, OS (HR 0.72 [95% CI 0.56‐0.93] *p* = 0.005]) (vs ChT+ placebo)
Pembrolizumab + carboplatin + paclitaxel → pembrolizumab	Primary advanced or recurrent **endometrial carcinoma**	NRG‐GY018 / KEYNOTE‐868 NCT03914612	06/2024	dMMR: mPFS NR vs. 6.5 mo (HR 0.30 [95% CI, 0.19–0.48] *p* <.0001]); pMMR: mPFS 11.1 vs. 8.5 mo (HR 0.6 [95% CI, 0.46–0.78] *p* <.0001]) (vs ChT+ placebo)
Pembrolizumab + gemcitabine + cisplatin	First‐line treatment of **biliary tract cancer**	KEYNOTE‐966 NCT04003636	10/2023	mPFS 6.5 vs. 5.6 mo, mOS 12.7 vs. 10.9 mo (HR 0.83 [95% CI, 0.72‐0.95] *p* = 0.0034) (vs ChT+placebo)
Pembrolizumab + pemetrexed + platinum	First‐line treatment of unresectable or metastatic **pleural mesothelioma**	KEYNOTE‐483 NCT02784171	09/2024	mPFS 7.1 mo both, mOS 17.3 vs. 16.1 mo (HR 0.79 [95% CI, 0.64‐0.98] *p* = 0.0162) (vs ChT+placebo)
Pembrolizumab + trastuzumab + chemotherapy	First‐line HER2‐positive **gastric/GEJ adenocarcinoma** (PD‐L1 CPS ≥1)	KEYNOTE‐811 NCT03615326	03/2025	mPFS 10 vs. 8.1 mo (HR = 0.73 [95% CI, 0.61‐0.87]), mOS 20 vs. 16.8 mo (HR 0.87 [95% CI, 0.67‐0.94] *p* = 0.004 *p* = 0.084) (vs SOC ChT + trastuzumab)
Pembrolizumab + chemotherapy	First‐line HER2‐negative **gastric/GEJ adenocarcinoma**	KEYNOTE‐859 NCT03675737	11/2023	mPFS 6.9 vs. 5.6 mo, mOS 12.9 vs. 11.5 mo (HR 0.78 [95% CI, 0.70‐0.87] *p* < .0001) (vs ChT+placebo)
Pembrolizumab peri‐operative (with RT ± cisplatin) in CPS ≥1	Resectable locally advanced **HNSCC**	KEYNOTE‐689 NCT03765918	06/2025	mEFS 51.8 vs. 30.4 (HR= 0.73 [95% CI, 0.58‐0.92], *p* = 0.0041) (vs SOC ± ChT+radiotherapy)
Nivolumab				
Nivolumab (adjuvant)	Adjuvant treatment of completely resected Stage IIB/C **melanoma**	CheckMate‐76K NCT04099251	10/2023	12‐mo RFS 89% vs. 79% (HR 0.42 [95% CI: 0.30–0.59] *p* < 0.0001), mRFS not reached (vs placebo)
Nivolumab + platinum‐doublet chemotherapy → adjuvant nivolumab	Peri‐operative treatment of resectable **NSCLC**	CheckMate‐77T NCT04025879	10/2024 (neo‐adj. only on 03/2022)	18‐mo EFS 70.2% vs. 50% (HR 0.68 [97.36% CI, 0.42 to 0.81] *p* < 0.001) (vs ChT+ placebo), mEFS not reached, pCR 25.4% vs. 4.7%, OR 6.64 [95% CI, 3.40 to 12.97] (vs SOC ChT)
Nivolumab + cisplatin + gemcitabine	First‐line treatment of unresectable or metastatic **urothelial carcinoma**	CheckMate‐901 NCT03036098	03/2024	PFS 7.9 mo vs. 7.6 mo (HR 0.72 [95% CI 0.59 to 0.88] *p* = 0.001), OS 21.7 mo vs. 18.9 mo (HR 0.78 [95% CI 0.63 to 0.96] *p* = 0.02), ORR 57.6% vs. 43.1% (vs. SOC ChT)
Others				
Cemiplimab (adjuvant)	Adjuvant treatment of **CSCC** at high risk of recurrence after surgery and RT	C‐POST NCT03969004	10/2025	68% lower recurrence/death risk (HR 0.32 [95% CI 0.20 to 0.51] *p* < 0.001), 24‐mo DFS 87.1% vs. 64.1% (HR 0.32 [95% CI, 0.20 to 0.51] *p* < 0.001) (vs placebo)
Cemiplimab + platinum‐based chemotherapy	First‐line treatment of advanced **NSCLC** without EGFR/ALK/ROS1 alterations	EMPOWER‐Lung 3 part 2, Study 16113 NCT03409614	11/2022	mPFS 8.2 vs. 5.5 mo (HR 0.55 [95% CI: 0.44‐0.68] *p* < 0.0001); mOS 21.1 vs. 12.9 mo (HR 0.65 [95% CI: 0.51‐0.82] *p* = 0.0003); ORR 43.6% vs. 22.1% (cemiplimab+chemo vs. chemo)
Retifanlimab (single agent)	Recurrent or metastatic **SCAC** after platinum therapy	POD1UM‐202 NCT03597295	05/2025	mPFS 2.3 mo [95% CI: 1.9‐3.6], mOS 10.1 mo [95% CI: 7.9‐NR], ORR 13.8% [95% CI: 7.6%–22.5%]
Retifanlimab + carboplatin + paclitaxel	First‐line treatment of inoperable recurrent or metastatic **SCAC**	POD1UM‐303 / InterAACT‐2 NCT04472429	05/2025	mPFS 9.3 mo vs. 7.4 mo (HR 0.63 [95% CI: 0.47‐0.84] *p* = 0.0006), mDOR 14.0 mo vs. 7.2 mo, mOS 29.2 vs. 23 mo (HR=0.7 CI: 0.40‐1.01] *p* = 0.0273) (vs ChT+placebo)
Penpulimab + cisplatin/carboplatin + gemcitabine	First‐line treatment of recurrent or metastatic **nasopharyngeal carcinoma**	AK105‐304 NCT04974398	04/2025	mPFS 9.6 mo vs. 7.0 mo (HR 0.45 [95% CI: 0.33–0.62] *p* < 0.0001), 12‐mo PFS 31% vs. 11%; OS data immature (vs ChT+placebo)
**Anti – PDL‐1**				
Atezolizumab (single agent)	Unresectable or metastatic **alveolar soft part sarcoma**	ML39345 (NCT03141684)	09/2022	12m DOR 42%, ORR 24% [95% CI: 13–39]
Atezolizumab + lurbinectedin (maintenance)	Maintenance treatment of Extended Stage **SCLC** without progression after induction atezolizumab + carboplatin + etoposide	IMforte NCT05091567	10/2025	mPFS 5.4 mo vs. 2.1 mo (HR 0.54 [95% CI: 0.43–0.67] *p* < 0.0001), mOS 13.2 mo vs. 10.6 mo (HR 0.73 [95% CI: 0.57–0.95] *p* = 0.0174) (vs. atezo only)
Cosibelimab (PD‐L1, single agent)	Metastatic or locally advanced **CSCC** not candidates for curative surgery or RT	CK‐301‐101 NCT03212404	12/2024	24‐mo DOR 72.1% (mCSCC), 80.2% (laCSCC), ORR 50.0% (mCSCC) [95% CI, 38%–62%], 54.8% (laCSCC) [95% CI, 36%–73%)]
Durvalumab (single agent)	Limited‐stage **SCLC** without progression after concurrent chemoradiation	ADRIATIC NCT03703297	12/2024	mPFS 16.6 mo vs. 9.2 mo (HR 0.76 [95% CI: 0.61–0.95] *p* = 0.0161), mOS 55.9 mo vs. 33.4 mo (HR 0.73 [95% CI: 0.57–0.93] *p* = 0.0104) (durvalumab vs. placebo)
Durvalumab + gemcitabine + cisplatin	First‐line treatment of unresectable or metastatic **biliary tract cancer**	TOPAZ‐1 NCT03875235	09/2022	mPFS 7.2 vs. 5.7 mo (HR 0.75 [95% CI: 0.63–0.89] *p* = 0.001), mOS 12.8 mo vs. 11.5 mo (HR 0.8 [95% CI: 0.66–0.97] *p* = 0.021) (vs SOC ChT only)
Durvalumab + platinum‐based chemotherapy (peri‐operative)	Resectable **NSCLC**	AEGEAN NCT03800134	08/2024	24‐mo EFS 63.3% vs. 52.4% (HR 0.68 [95% CI 0.53–0.88] *p* = 0.004) (vs SOC ChT only)
Durvalumab + gemcitabine + cisplatin → durvalumab	Peri‐operative treatment of resectable **muscle‐invasive bladder cancer**	NIAGARA NCT03732677	03/2025	24‐mo EFS 67.8% vs. 59.8% (HR 0.68 [95% CI: 0.56–0.86] *p* < 0.001); 24‐mo OS 82.2% vs. 75.2% (HR 0.75 [95% CI 0.59, 0.93] *p* = 0.01)
**Immune Checkpoint Inhibitor Doublets**				
Nivolumab + ipilimumab	First‐line treatment of unresectable or metastatic **HCC**	CheckMate‐9DW NCT04039607	04/2025	DOR 30.4 mo vs. 12.9 mo, mOS 23.7 mo vs. 20.6 mo (HR 0.79 [95% CI: 0.65–0.96] *p* = 0.018)
Nivolumab + ipilimumab	First‐line treatment of advanced **ESCC**	CheckMate‐648 NCT03143153	05/2022	mPFS not superior to chemo, mOS 12.8 mo vs. 10.7 mo (HR 0.78 [95% CI: 0.65‐0.93 *p* = 0.01) in nivo‐contatining regimen vs. ChT only, in all‐comers (Arms: ipilimumab nivolumab, nivolumab+ChT, ChT)
Durvalumab + tremelimumab	Unresectable **HCC**	HIMALAYA NCT03298451	10/2022	mPFS NS, mOS 16.4 mo vs. 13.8 mo (HR 0.78 [96.02% CI 0.65–0.93] *p* = 0.0035), 5‐yr OS 19.6% vs. 9.4% (vs sorafenib)
Relatlimab + nivolumab	Unresectable or metastatic **melanoma**	RELATIVITY‐047 NCT03470922	03/2022	mPFS 10.2 mo vs. 4.6 mo (HR 0.79 [95% CI, 0.66–0.95]), numerical benefit only ‐ mOS 51.0 mo vs. 34.1 mo (HR 0.80 [95% CI, 0.66–0.99], initial *p* = 0.059) (vs. nivolumab)

MSI = Microsatellite Instability, dMMR‐ deficient mismatch repair, pMMR‐ Proficient mismatch repair, PFS = Progression free survival, RFS = Recurrence free survival, mDOR = median duration of response, EFS = Event free survival, mOS = Median overall survival, DMFS = distant metastasis‐free survival, ChT = Chemotherapy, NSCLC = Non‐small cell lung cancer, GEJ = Gastroesophageal junction, HNSCC = Head and neck squamous cell carcinoma, CSCC = Cutaneous squamous cell carcinoma, SCAC = Squamous cell carcinoma of the anal canal, SCLC = Small cell lung carcinoma, ESCC = Esophageal squamous cell carcinoma, HCC = Hepatocellular carcinoma

Several additional immune co‐inhibitory molecules have been studied. T‐cell immunoglobulin and mucin‐domain–containing 3 (TIM3) was first identified in 2002 as a transmembrane protein expressed on CD4+ Th1 cells and CD8+ Tc1 cells, and was later found to be expressed also on regulatory T (Treg) cells, dendritic cells (DCs), natural killer (NK) cells, monocytes, macrophages, and mast cells [102]. TIM3 cooperates with several ligands to initiate its negative regulatory functions, including Galectin‐9, carcinoembryonic antigen‐related cell adhesion molecule 1 (CEACAM1), phosphatidylserine (PtdSer), and high‐mobility group protein B1 (HMGB1). Its expression is associated with T‐cell dysfunction, as demonstrated in mouse‐model TILs that exhibit the most severe exhausted phenotype with failure to proliferate and produce IL‐2, TNF, and IFN‐γ [103]. It is also highly expressed on Foxp+ Tregs, especially within the tumor microenvironment rather than in peripheral blood [104]. Several solid tumors have high expression of TIM‐3, and high levels are negatively prognostic for survival [105, 106, 107]. Anti‐TIM‐3 antibodies are currently being investigated in phases clinical trials, such as the AMBER study [108, 109], mainly in combination with anti‐PD‐1. The study revealed acceptable safety profile and preliminary antitumor activity, with efficacy most notable in immunotherapy‐naïve melanoma and hepatocellular carcinoma, and modest activity in PD‐1/PD‐L1‐refractory non‐small cell lung cancer.

T‐cell immunoreceptor with Ig and ITIM domains (TIGIT), is another broadly studied inhibitory checkpoint expressed on activated T‐cells, regulatory T‐cells, and natural killer (NK) cells. TIGIT exerts its immunosuppressive activity by competing with CD226 (DNAM‐1) and CD96 for engagement with their shared ligands—primarily CD155 (PVR) and CD112—on APCs and tumor cells. As this competitive axis closely parallels the CTLA‐4/B7/CD28 pathway, therapeutic blockade of TIGIT may similarly restore co‐stimulatory signaling and offer clinical benefit [110].

Tiragolumab, a fully human IgG1/kappa anti‐TIGIT mAb, inhibits the interaction between TIGIT and CD155 [111]. Although TIGIT was considered a promising immunotherapy target, phase III trials combining tiragolumab with anti‐PD‐L1 were unsuccessful [112]. The failure was partially attributed to the Fc‐active anti‐TIGIT antibodies, which trigger ADCC against TIGIT^+^ exhausted T‐cells and Tregs but also induce depletion of activated TIGIT^+^ NK cells, ultimately impairing NK‐cell antitumor function [113]. Fc‐silent anti‐TIGIT antibodies maintain NK‐cell function, whereas Fc‐active versions impair it by depleting TIGIT^+^ NK cells. Improving specificity can be approached through several strategies currently under investigation, including the development of bispecific antibodies that preferentially target exhausted T‐cells [114, 115] and the engineering of Fc‐silenced antibody domains [116]. This experience highlights that failure of checkpoint combinations may not be related to inadequate target biology, but also from antibody engineering and effects on non‐T‐cell immune populations, supporting development of Fc‐silent or selective targeted TIGIT‐based agents.

Antibodies activating immune co‐stimulatory molecules have also been studied, and results to date have been disappointing. The most extensively studied include those belonging to the immunoglobulin super‐family, activators of CD28, ICOS, CD226 or CRTAM) and TNF receptor super‐family, including 41‐BB, OX40, CD27, GITR, HVEM, CD40, BAFFR, BAFF and others [117].

## 3 Antibody Based Therapies

### 3.1 Antibody‐Based Immune Modulators

The definition of immunotherapy is opaque when it comes to antibodies that bind to tumor cells resulting in antibody‐dependent cellular cytotoxicity (ADCC) or antibody‐dependent cellular phagocytosis (ADCP). Antibodies are structurally composed of several domains—the fragment antigen‐binding (F(ab)) region, the fragment crystallizable (Fc) region, which interacts with Fc receptors (FcRs), and the hinge region. The Fc portion enables direct engagement with Fcγ‐receptors (FCGR) on various immune cells, allowing monoclonal antibodies to activate multiple immune responses, including ADCC, ADCP, and CDC [118]. Use of tumor cell targeting antibodies has advanced substantially in recent years, with the approval of new specific unconjugated antibodies [119], bispecific tumor‐targeting antibodies [120] and biparatopic antibodies that bind two regions of the same antigen [121].

### 3.2 Engineered Antibody‐Based Immune Modulators – Bispecific T‐Cell Engagers

Bispecific antibodies (BsAbs) are engineered antibodies that can simultaneously bind to two different epitopes of an antigen or two different antigens. Bispecific T‐cell engagers (BiTEs) bind both a tumor‐associated antigen and the CD3 subunit within the T‐cell receptor (TCR) complex, thereby recruiting T‐cells to the tumor and redirecting cytotoxicity toward malignant cells. By engaging both targets simultaneously, BiTEs promote the formation of an artificial cytolytic synapse that resembles physiologic T‐cell‐mediated lytic synapses, leading to polyclonal T‐cell activation, granule polarization, and perforin‐ and granzyme‐dependent tumor cell lysis [122].

MHC‐I downregulation is a common mechanism of immune evasion, impairing recognition by endogenous CD8+ T‐cells and contributing to resistance to immune checkpoint blockade [123]. Due to their mechanism of action, BiTEs bypass the need for antigen recognition via MHC class I or II molecules, as well as the involvement of APCs or costimulatory molecules [124]. This may allow BiTEs to retain activity in tumors with impaired antigen presentation, as preclinical models have shown that they remain active despite MHC class I loss and can partially restore T‐cell effector function [123]. However, additional evidence suggests that MHC‐I may still contribute to optimal BsAb efficacy in some settings, because MHC‐I‐dependent signaling can further enhance BsAb‐induced CD8+ T‐cell expansion and effector differentiation [125]. Therefore, although BsAbs do not require MHC‐I for primary cytotoxicity, preserved MHC‐I expression may still improve the depth and durability of response.

There has been significant progress in the development of BiTEs in hemato‐oncology, likely due to the greater availability of target molecules expressed on the cells surface with sufficient tumor selectivity to avoid on‐target off‐tumor toxicity [124, 125]. Several factors have limited the applicability of BiTEs in solid tumors. Compared with the high and relatively uniform expression of lineage‐restricted antigens in hematologic malignancies, surface antigens in solid tumors are often less tumor‐specific, more heterogeneously expressed, and in some cases present at densities insufficient for efficient immune synapse formation [126, 127]. In addition, abnormal vasculature and dense stromal architecture act as physical barriers in solid tumors, impairing antibody distribution and limiting T‐cell access to malignant cells [126]. These limitations are further amplified by the profoundly immunosuppressive microenvironment of solid tumors. Multiple factors contribute to this hostile setting, including infiltration by suppressive cellular populations, inhibitory soluble mediators, and adaptive upregulation of immune checkpoints, which collectively promote progressive dysfunction and exhaustion of cytotoxic T‐cells [126, 127].

**FIGURE 5:**
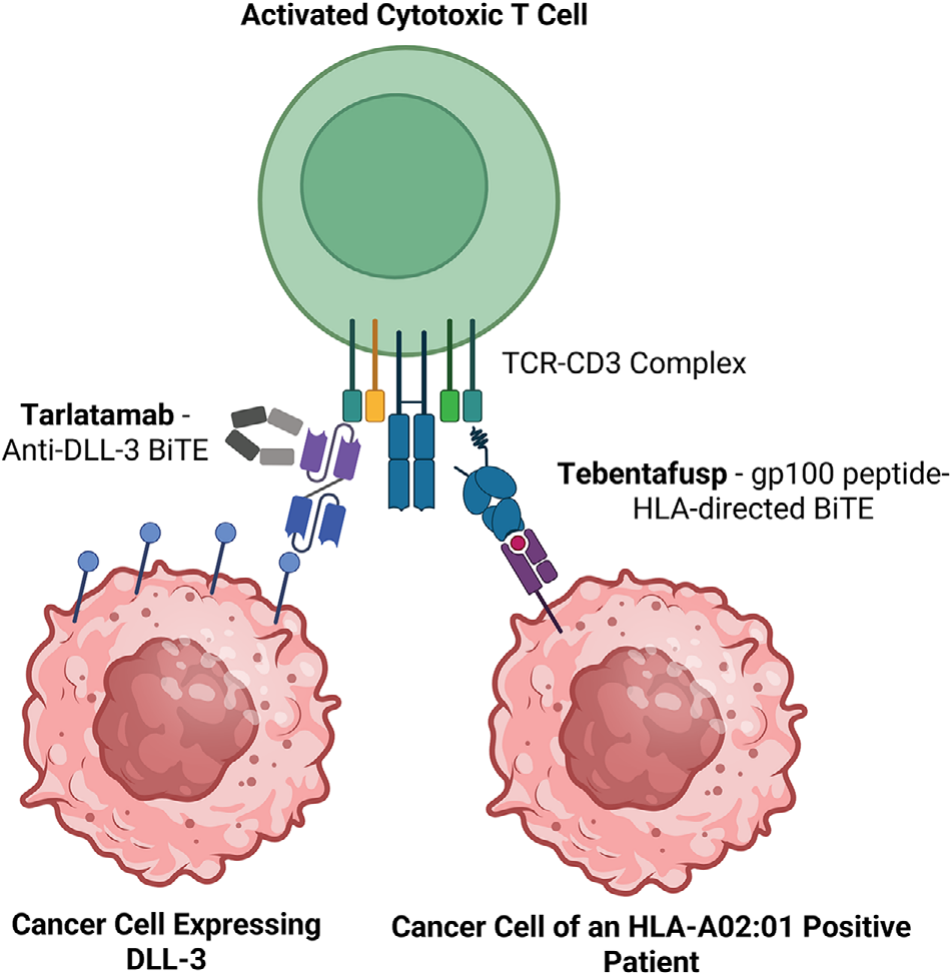
Structure and mechanisms of action of approved bispecific T‐cell engagers (BiTEs) for solid tumors. BiTEs are bispecific antibodies designed to physically link cytotoxic T cells to tumor cells. One arm binds the CD3 component of the T‐cell receptor complex, while the other recognizes a tumor‐associated antigen or peptide–HLA complex on cancer cells, leading to T‐cell activation, immune synapse formation, and antigen‐dependent tumor cell killing. Approved agents include tarlatamab, targeting DLL3‐expressing tumor cells, and tebentafusp, which recognizes gp100 peptide presented by HLA‐A*02:01 in eligible patients.

To date there are two approved BiTEs for solid tumors, tebentefusp and tarlatamab (Figure 5). Because traditional bispecific antibodies can target only surface‐expressed proteins, antibodies that recognize intracellular protein–derived peptides presented on HLA molecules have been developed, thereby expanding the range of tumor‐associated antigens that can be targeted. However, this approach is limited to patients who carry the specific HLA allele presenting the targeted peptide, the most common being HLA‐A*0201 [128]. Tebentafusp, a bispecific fusion protein that recognizes CD3 and a peptide fragment of gp100 presented by HLA‐A*0201 molecules on tumor cells, was the first BiTE to receive FDA approval in 2022, for the treatment of HLA‐A*0201‐positive patients with unresectable or metastatic uveal melanoma [129]. Approximately 45% of the population of the USA and Europe are HLA‐A*0201‐positive. Tebentefusp demonstrated a statistically significant improvement in survival despite a modest response rate of 9%, possibly due to the fact that commonly used radiographic treatment response assessment criteria such as the Response Evaluation Criteria in Solid Tumors (RECIST) may not account for response patterns associated with immune activation and shrinkage of infiltrative liver masses [129]. It is possible that ctDNA will become a more sensitive marker of clinical benefit than radiographic response [130].

The second approved bispecific molecule is tarlatamab, a bispecific antibody targeting delta‐like ligand 3 (DLL3), highly expressed on SCLC cells, and CD3 on T‐cells. A recent major development in the therapy of SCLC is due to the addition of tarlatamab to the standard of care. The DeLLphi‐304 phase 3 trial [131] randomized patients with relapsed SCLC to receive tarlatamab or standard chemotherapy (topotecan, lurbinectedin, or amrubicin), showing a mOS of 13.6 months with tarlatamab versus 8.3 months with chemotherapy, establishing tarlatamab as a new second‐line standard after progression on platinum‐based therapy. Tarlatamab in combination with PD‐L1 inhibitors as first‐line maintenance following chemo‐immunotherapy is being evaluated now in a phase 1‐b trial [132], recently published an incredible primary mOS of more than 25 months. It seems that tarlatamab has the potency to transition SCLC into a hot tumor, with an obvious increase in sensitivity to immunotherapy.

In addition to representing a major change in standard care for SCLC, there are currently other clinical trials testing tarlatamab in aggressive neuroendocrine prostate cancer [133] and in other neuroendocrine DLL‐3 positive tumors in a phase 2 basket trial [134].

Efforts are ongoing to develop BiTEs targeting PSMA, a very specific surface protein of prostate cancer. JANX007 is a humanized trispecific protein that contains PSMA and CD3‐binding domains, an albumin binding domain to extend circulating half‐life, and a CD3 inhibitory peptide mask fused to the molecule through a tumor protease cleavable linker [135]. It has a tumor specific cleavable mechanism, and the active molecule can bind CD3 only once tumor‐resident proteases cleave the protein and enable mask separation. This could potentially limit systemic toxicity associated with broad T‐cell activation and is currently in phase I/Ib trials.

Treatment with bispecific antibodies leads to T‐cell activation, degranulation, and release of proinflammatory cytokines, which can trigger cytokine release syndrome (CRS) and immune effector cell‐associated neurotoxicity syndrome (ICANS). Both toxicities appear early—most often within the first 24 hours after the infusion [136]. CRS presents mainly with symptoms ranging from fever to severe hypoxia and hypotension. Step‐up or intermittent dosing results in a decrease in cytokine responses, and CRS becomes less frequent with subsequent administration. Supportive management includes antipyretics, fluids, and oxygen, and if needed – steroids, IL‐6 receptor blockade. Most patients experience rapid clinical improvement, although severe cases may require ICU monitoring. Importantly, the incidence and severity of CRS are lower with BiTE therapies relative to chimeric antigen receptor T‐cell therapies. Grade 1–2 CRS is relatively common, but in the DeLLphi‐301 trial there were no grade 4–5 events and only one grade 3 event [136]. ICANS is far less common, especially in the solid tumors setting, and it is mainly diagnosed by exclusion [137]. Symptoms can resemble those of many other diseases, such as infection/sepsis, stroke, paraneoplastic syndromes, pain medication use, and new or progressive disease in the central nervous system [138].

### 3.3 Antibody‐Drug Conjugates

The rationale for combining chemotherapy with immunotherapy is to achieve synergistic antitumor effects by utilizing complementary mechanisms of action. Although chemotherapy is generally effective, eventually escape and resistance frequently occur in residual malignant cells at tolerated doses. However, chemotherapy can induce immunogenic cell death, releasing tumor antigens and damage‐associated molecular patterns (DAMPs) that enhance antigen presentation and activate both innate and adaptive immune responses [139]. This process can increase tumor immunogenicity and improve immune recognition, thereby priming the tumor microenvironment for concurrent or subsequent immunotherapy [140, 141]. Some even suggested developing a method of immune induction mediated by short‐term chemotherapy, as in the TONIC trial with doxorubicin and cisplatin [142].

The combination of chemotherapy and immunotherapy has demonstrated benefit in several settings, such as in neo‐adjuvant treatment for triple‐negative breast cancer, a highly aggressive disease with high rates of recurrence for patients without pCR after pre‐operative treatment [143]. Perioperative chemo‐immunotherapy is as effective in patients with loss of HLA class I expression and loss of heterozygosity, that tend to have lack of response to immunotherapy [144].

**FIGURE 6:**
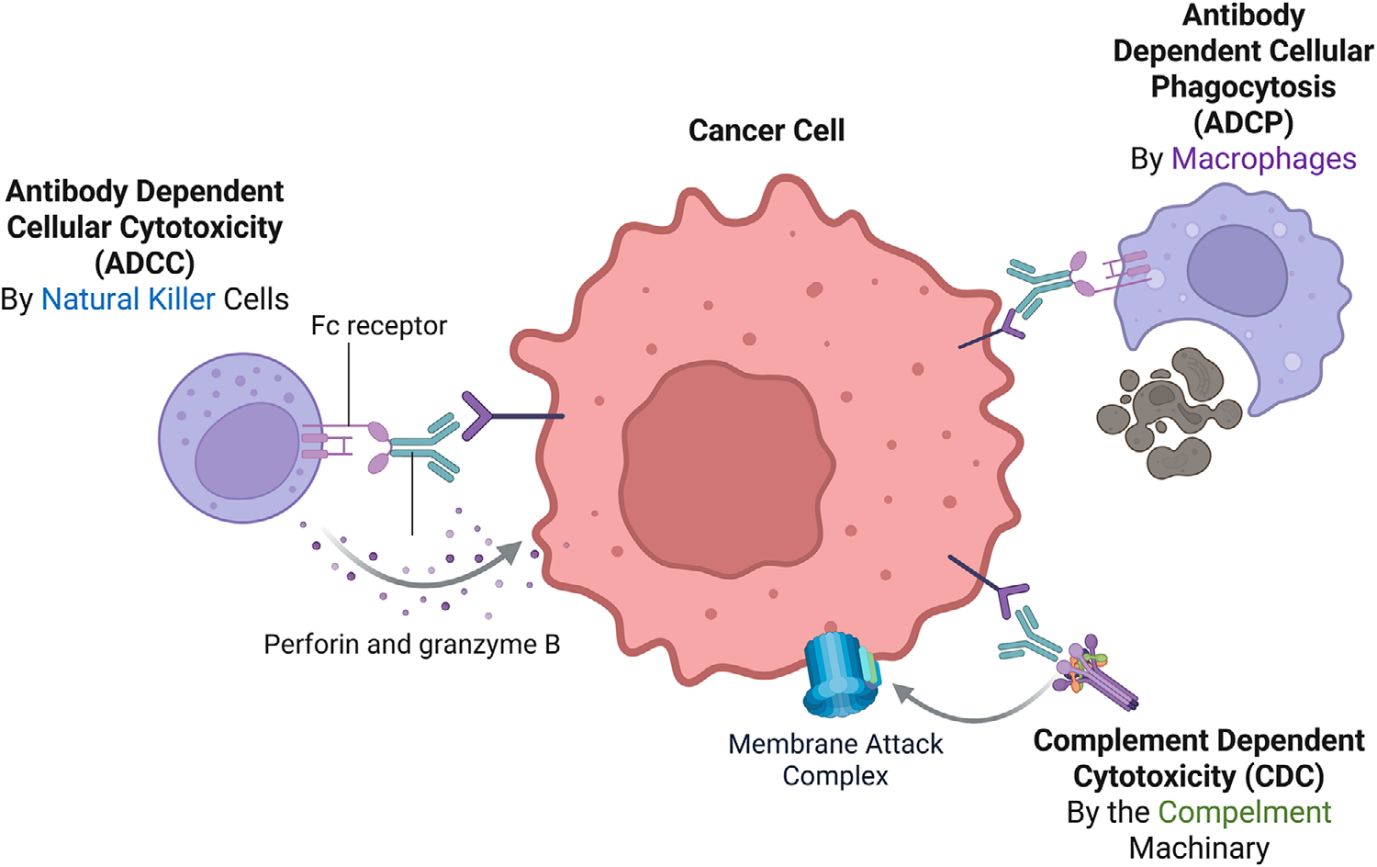
Different mechanisms of antibody‐dependent cytotoxicity. Therapeutic antibodies eliminate tumor cells through Fc‐mediated effector functions, including antibody‐dependent cellular cytotoxicity by natural killer cells, antibody‐dependent cellular phagocytosis by macrophages, and complement‐dependent cytotoxicity via activation of the complement cascade.

It was therefore logical to subsequently evaluate the combination of antibody–drug conjugates (ADCs) with immune checkpoint inhibitors. ADCs consist of a tumor‐specific antibody that binds to the target cell antigen, as with any monoclonal antibody, a linker, and a cytotoxic payload, as in chemotherapy. This structure permits multiple mechanisms of action, including ADCC, ADCP, and CDC, as well as immunogenic cell death resulting from neo‐antigens released following the direct cytotoxic effect of the payload (Figure 6). Tumor‐associated macrophages (TAMs) may also participate in a Fc‐FcɣR interaction, potentially releasing the cytotoxic payload within the tumor microenvironment (TME), creating a bystander effect that may improve response in heterogeneous tumors but can also theoretically decrease the efficacy of immunotherapy by eliminating active T‐cells within the tumor [145, 146].

The ability of ADCs to promote antitumor immunity appears to vary substantially across agents and is shaped by the interplay between payload biology and ADC architecture, which together determine the extent to which tumor cell killing is coupled to immune priming. Among these variables, the payload appears to be a major determinant of whether ADC‐induced cytotoxicity is accompanied by immunogenic cell death and broader activation of innate immune pathways.

Topoisomerase I inhibitor payloads have emerged as especially attractive partners for immune checkpoint blockade, as they can promote immunogenic cell death and, beyond direct cytotoxicity, generate DNA damage and micronuclei that activate the cGAS‐STING pathway, leading to type I interferon signaling and contributing to a more inflamed tumor microenvironment [147]. Together with the bystander effect associated with membrane‐permeable payloads, these features may broaden antigen release and enhance antitumor immune priming, supporting the rationale for combining select ADCs with PD‐1/PD‐L1 blockade [148].

Over the past few years a few ADC‐ICI combinations have been tested. The combination of enfortumab vedotin (Nectin‐4‐ directed ADC delivering MMAE, microtubule‐disrupting agent monomethyl auristatin E) and pembrolizumab is now established as a preferred first‐line treatment for patients with metastatic urothelial carcinoma [149]. This approval is based on the results of the phase 3 EV‐302/KEYNOTE‐A39 trial, which demonstrated that the combination significantly improved PFS (12.5 months vs. 6.3 months; HR 0.45) and OS (31.5 months vs. 16.1 months; HR 0.47) compared to platinum‐based chemotherapy. This represents a dramatic change in prognosis for all bladder cancer patients, but particularly for the cisplatin‐ineligible population, which previously had very limited options. This approach has also been tested in metastatic triple‐negative breast cancer. In the past few years, for patients with PD‐L1 expressing tumors, the standard of care included first‐line chemotherapy and pembrolizumab [150]. However, the standard of care may change to ADC combined with anti‐PD‐1 as a result of the ASCENT‐04/KEYNOTE‐D19 trial demonstrating a statistically significant improvement in PFS with the combination of sacituzumab govitecan (Trop‐2–directed ADC delivering SN‐38, a topoisomerase I inhibitor) and pembrolizumab [151].

PDL‐1 targeted ADCs are an interesting approach, aimed at blocking the PD‐1/PD‐L1 interaction and T‐cell inhibition, while simultaneously inducing a cytotoxic effect in the tumor [152]. A critical challenge in the PDL‐1 ADC design lies in the dual targeting of both tumor cells and PDL‐1‐expressing T‐cells or macrophages, which may increase the risk of off‐tumor toxicity and unintended immune modulation. Possible solutions may include linkers with a selective cleavage initiated in the tumor microenvironment, tumor‐selective payloads, and their combination with immunostimulatory agents. Tumor antigen heterogeneity, including variable PDL‐1 expression across different cancer types and within tumor microenvironments, may also complicate uniform targeting and payload delivery. This field is evolving, with some novel drugs being tested in pre‐clinical models and early phases clinical trials [153, 154, 155].

## 4 Advanced Cellular and Immune‐Activating Therapies

**FIGURE 7:**
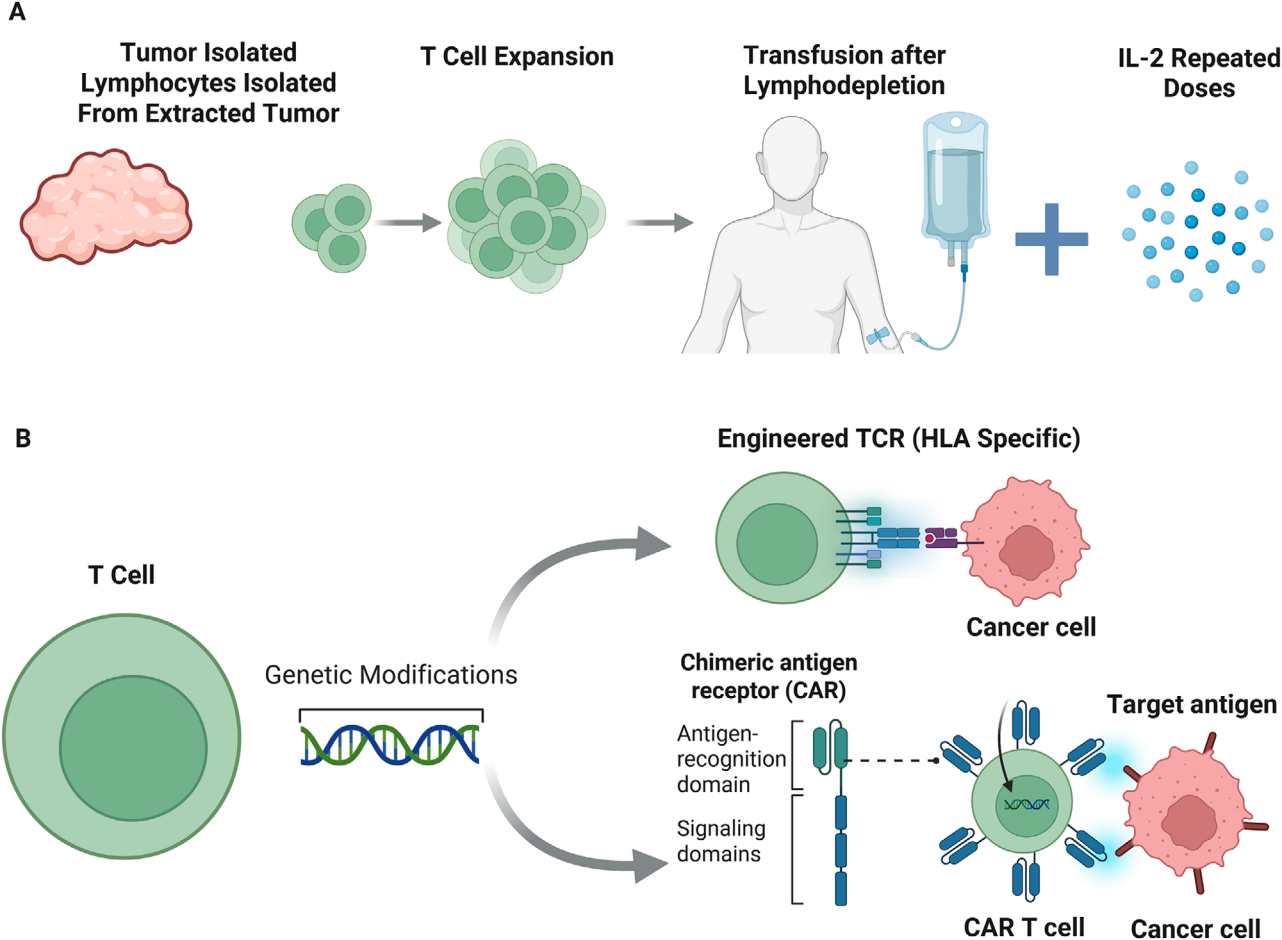
Mechanisms of action of TIL, TCR‐T, and CAR‐T therapies (A) Tumor‐infiltrating lymphocytes (TILs) are isolated from resected tumor tissue, expanded ex vivo, and reinfused following lymphodepleting chemotherapy, with interleukin‐2 supporting in vivo T‐cell persistence and activity. (B) TCR‐T and CAR‐T therapies involve genetic modification of T cells to express engineered antigen receptors. TCR‐T cells recognize intracellular tumor antigens presented by specific HLA molecules, whereas CAR‐T cells target surface antigens in an HLA‐independent manner, leading to direct tumor cell killing.

Advances in immunobiology have led to the emergence of therapeutic modalities that extend beyond checkpoint inhibition and conventional antibody‐based approaches. Adoptive cell therapies—including tumor‐infiltrating lymphocyte (TIL) therapy, chimeric antigen receptor (CAR) T‐cells, and T‐cell receptor (TCR)–engineered T‐cells (Figure 7)—seek to increase the number and functional competence of effector T‐cells capable of recognizing tumor antigens. In parallel, immune‐stimulatory interventions such as cytokine‐based therapies and cancer vaccines attempt to enhance or initiate tumor‐specific immunity through non–cell‐transfer mechanisms. Together, these approaches represent a broad spectrum of next‐generation immunotherapies with the potential to address limitations observed with existing treatments.

TIL therapy is an adoptive cell transfer modality most extensively employed in melanoma [156]. The treatment requires surgical excision of a tumor deposit from which TILs are isolated and subsequently expanded in culture over several weeks. After completion of a lymphodepleting chemotherapy regimen, the expanded lymphocytes are reinfused, followed by administration of high‐dose interleukin‐2 (IL‐2) to promote their survival and proliferation. The resulting product is a polyclonal population of tumor‐reactive T‐cells that recognize diverse tumor antigens through their endogenous T‐cell receptors, enabling direct cytotoxic activity and functional shaping of the tumor microenvironment.

Lifileucel is currently the only FDA‐approved autologous TIL therapy in solid tumors, approved for patients with metastatic melanoma that had previously progressed on PD1/L1 inhibitors. Approval was granted based on the phase 2 C‐144‐01 trial [157]. The ORR was 31.4% and 79.3% of patients had overall tumor burden reductions. Of note, not all tumor reduction was durable, likely indicative of transient tumor shrinkage due to chemotherapy. The mOS was 13.9 months with 5‐year OS rate of 19.7% [158]. The treatment has relatively significant toxicity, with 7.7% deaths due to AEs (12 patients), most of them attributed to lymphodepletion. Ongoing trials suggest the possibility that the benefit from TIL therapy in the melanoma setting may lie in ICI‐naïve patients [159]. There are also other developments in different types of cancer, such as cervical cancer with T‐cell selection for E6 and E7 reactivity (HPV‐TILs) [160].

Ongoing efforts to modify TIL therapy and decrease the morbidity include OBX115, a product that has received fast track designation by the FDA based on promising early results, as reviewed [161]. Specifically, OBX115 is an engineered TIL product armored with membrane bound IL15 that can be induced with oral acetazolamide. This obviates the need for high‐dose IL2 after TIL infusion, avoiding cytokine‐related symptoms associated with high dose IL2, as the IL15 release is not systemic. Moreover, the TIL product can be generated from core biopsies rather than surgical resection as is necessary for Lifileucil. It is being studied in melanoma and non‐small cell lung cancer. Other approaches to modify TIL and decrease morbidity include combinations with immune checkpoint inhibitors, metabolic reprogramming of TIL, and additional genetic modifications to enhance TIL survival, as reviewed [162].

Modified T‐cell‐based therapies have dramatically reshaped the management of hematologic cancers. CARs are synthetic fusion proteins composed of an extracellular antigen‐binding domain—usually a single‐chain variable fragment from an antibody, a transmembrane domain, and an intracellular signaling domain. CAR T‐cells can target any cell expressing the relevant surface antigen, regardless of the patient's HLA type, but are limited to antigens expressed on the cell surface. Most clinical successes in this area have come from products targeting CD19 or B‐cell maturation antigen (BCMA). Since 2017, the FDA has granted approval to numerous CAR T‐cell therapies for leukemias, lymphomas, and multiple myeloma across adult and pediatric populations [163]. However, the impressive outcomes seen in hematologic malignancies have not been consistently reproduced in solid tumors.

Among the most advanced programs for CAR‐T in solid tumors are CAR‐T‐cells directed against Claudin 18.2 in gastrointestinal malignancies, GD2 in neuroblastoma, and CD70 in renal cell carcinoma and other CD70‐expressing tumors. Early studies of Claudin 18.2–targeted CAR‐T‐cells in advanced GEJ/gastric have demonstrated ORR and disease control rate of 57.4% and 83.0%, respectively [164, 165]. GD2‐directed CAR‐T‐cells have produced high ORR, including complete responses, in pediatric neuroblastoma [166, 167]. CD70 is an attractive target due to its high expression on clear cell renal cell carcinoma cells, with CD70‐directed CAR‐T products demonstrating early signs of antitumor activity with acceptable safety profiles [168].

The comparatively limited efficacy of CAR T‐cell therapy in solid malignancies is widely attributed to features of the solid tumor, including a highly suppressive microenvironment, antigenic heterogeneity, and immune‐evasion mechanisms that collectively impair T‐cell activity [169]. To overcome these barriers, additional cellular approaches beyond CAR T‐cells have been explored. One such strategy is TCR T‐cell therapy, in which autologous T‐cells are genetically engineered ex vivo to express a high‐affinity TCR that recognizes a tumor‐associated peptide presented by a specific HLA allele [163]. This platform leverages the inherent T‐cell capability to recognize antigens displayed on HLA molecules. After infusion, these TCR‐T‐cells recognize intracellular or surface tumor antigens presented on HLA molecules, resulting in antigen‐specific T‐cell activation followed by cytokine release, proliferation, and targeted cytotoxicity against tumor cells. Unlike TIL therapy, HLA restriction is essential for deriving benefit from this treatment.

At present, TECELRA (afamitresgene autoleucel) tested in the SPEARHEAD‐1 phase 2 trial [170], is the only FDA‐approved engineered TCR‐T therapy, for adults with unresectable or metastatic synovial sarcoma who have been previously treated with chemotherapy. It is indicated for tumors that are MAGE‐A4 positive and individuals with HLA‐A*02:01, ‐A*02:02, ‐A02:03, or ‐A02:06. Approval was based on an ORR of 39% and a median duration of response of 11.6 months. Severe cytopenias were very common (81%–96%) but no treatment‐related deaths occurred in the study.

Ongoing trials are evaluating TCR T‐cell therapies targeting additional solid‐tumor antigens such as PRAME and NY‐ESO‐1. PRAME‐directed products have demonstrated manageable toxicity, objective responses in the range of ∼51%–57%, and tumor reduction in ∼89% across multiple tumor types [171], leading to phase III trials. New York esophageal squamous cell carcinoma oncoprotein 1 (NY‐ESO‐1) is a promising target, highly expressed in melanoma, multiple myeloma, and esophageal squamous cell carcinoma. Letetresgene autoleucel (lete‐cel) is an autologous T‐cell receptor therapy targeting NY‐ESO‐1 for patients with patients with advanced sarcoma who are HLA‐A*02:01 positive [172]. It has shown 40% RR in the phase II IGNYTE‐ESO trial in advanced synovial sarcoma and myxoid round cell liposarcoma, with a median response duration of 10.6 months, supporting continued development. Additional TCR candidates under study include targets such as HPV E7 [173], KRAS G12V and G12D [174, 175].

Taken together, these emerging modalities highlight the rapidly growing landscape of next‐generation immunotherapies beyond checkpoint blockade. Although TIL therapy, CAR T‐cells, and TCR‐engineered T‐cells are each associated with unique biological challenges, they collectively aim to expand the range of tumors that can be effectively targeted by cellular therapies. Ongoing advances in cellular engineering and combinations will be critical for fully unlocking the therapeutic potential of adoptive cell therapy in malignancies that have long remained resistant to immune‐based treatments.

## 5 Immune‐Modulating Systemic Therapies

### 5.1 Cytokine‐Based Therapies

Cytokine‐based therapies represent some of the earliest attempts to harness the immune system against cancer, and interleukin‐2 (IL‐2) remains the primary example of this approach. As a central T‐cell growth factor produced by activated T‐cells, IL‐2 drives proliferation, differentiation, and functional activation of multiple immune subsets—including CD8^+^ effector T‐cells and NK cells—while simultaneously expanding immunosuppressive regulatory T‐cells (Treg) through its high‐affinity trimeric receptor [176]. These dual and often competing immunologic effects have shaped both the benefits and the limitations of IL‐2 as anticancer therapy. High‐dose IL‐2 was the first immune‐stimulating cytokine to demonstrate durable tumor regression in metastatic melanoma and renal cell carcinoma, establishing a landmark proof‐of‐concept for immunotherapy decades before immune checkpoint inhibitors [1]. However, its clinical use has been constrained by a narrow therapeutic window, a short serum half‐life (5–7 minutes), and severe toxicities such as capillary leak syndrome, necessitating administration in highly specialized inpatient settings [177]. Due to its dual effect, it seems that one of the main resistance mechanisms to IL‐2 treatment is expansion of Tregs, resulting in limited anti‐tumoral activity [178, 179]. Today, high‐dose IL‐2 is being used after TIL infusion to promote the survival and proliferation of the T‐cell product, with fewer doses comparing to IL‐2 monotherapy [177].

Understanding these biological and clinical challenges provides the foundation for the development of next‐generation cytokines‐based agents, fusion proteins, and cytokine‐modulating strategies.

Recent advances focus on engineered IL‐2 variants and IL‐2 receptor agonists designed to selectively stimulate CD8^+^ T‐cells and NK cells while limiting Treg activation and reducing systemic toxicity. Multiple strategies—including pegylation, fusion‐protein constructs, antibody–cytokine fusion proteins (immunocytokines) and mutationally altered IL‐2 proteins (“muteins”)—have all been developed to enhance pharmacokinetics and improve tumor‐specific immune responses [176, 180, 181, 182].

AB248 (Etakafusp alfa) is an example to an engineered IL‐2, consisting of a fusion of attenuated IL2 mutein and an antibody designed for cis‐targeting of CD8β to preferentially activate CD8+ T‐cells over NK cells and Tregs. Pre‐clinical data demonstrated that the expected IL‐2 gene signature emerges following stimulation of CD8+T‐cells in vitro with AB248, and that AB248 prevents NK‐cell driven, non‐specific cytokine secretion by human PBMCs [183]. Based on this promising preclinical evidence, an ongoing phase 1a/b study is evaluating AB248 in a dose‐escalation and expansion design in patients with locally advanced or metastatic tumors that have progressed on prior therapies.

Other cytokines with potentially less pleiotropic cellular effects are being studied, and these include engineered and targeted approaches using interleukin‐15 (IL‐15), interleukin‐12 (IL‐12), interleukin‐18 (IL‐18), interleukin‐21 (IL‐21), interferons (especially IFN‐α and IFN‐γ), and granulocyte‐macrophage colony‐stimulating factor (GM‐CSF), as well as strategies to neutralize immunosuppressive cytokines such as TGF‐β [184]. An overview of the primary effects of key cytokines on the immune system is provided in Table 4.

**TABLE 4:** Primary effects of key cytokines on the immune system.

Cytokine	Dominant signaling pathways	CD8 T cells (effector / memory)	Tregs	NK cells	CD4 T cells	Overall inflammatory profile
IL‐2	JAK1/3–STAT5, PI3K–AKT	↑	↑	↑	↑	Both pro‐inflammatory (effector T/NK) and immunoregulatory (Treg)
IL‐12	JAK2–STAT4	↑	—	↑	↑	Strongly pro‐inflammatory
IL‐15	JAK1/3–STAT5, PI3K–AKT	↑	—	↑	↑	Pro‐inflammatory
IL‐18	MyD88–NF‐κB, MAPK	↑ (with IL‐12), less on memory	—	↑	↑	Both pro‐inflammatory (Th1, NK) and can promote type 2 inflammation
IL‐21	JAK1/3–STAT3	↑	↓	↑	↑ (Tfh, Th17)	Both pro‐inflammatory (Th17) and immunoregulatory

↑ increase; ↓ decrease; – no dominant effect.

IL‐15 shares many biological properties with IL‐2, and it can also increase the proliferation of NK and T‐cells in peripheral tissues. Moreover, IL15Rα is not expressed on Treg cells, which is one of the differences between IL‐15 and IL‐2, and protects IL15 from Treg‐mediated suppression. IL‐15 does require trans‐engagement through IL15Rα subunit on myeloid and lymphoid cells, and for that reason IL15/IL15Rα super‐agonistic fusion proteins were developed, with interesting pre‐clinical results and attempts in early‐phase clinical trial [185].

IL‐12 is a heterodimeric cytokine mainly produced by dendritic cells, monocytes, macrophages, and B cells, and plays a central role in promoting antitumor immune responses. By binding to the IL‐12 receptor (IL‐12Rβ1/β2) on T‐cells and NK cells, IL‐12 activates the JAK–STAT pathway, resulting in robust interferon‐γ production, Th1 differentiation of CD4^+^ T‐cells, and enhanced cytotoxic activity of CD8^+^ T‐cells and NK cells(187). Early clinical studies with systemic recombinant IL‐12 were limited by insufficient efficacy and significant toxicity; however, major advances have been achieved through localized and tumor‐targeted delivery strategies. These include intratumoral gene‐based approaches using viral or non‐viral vectors, as well as mRNA delivery platforms such as lipid nanoparticle‐based formulations, which enable high local IL‐12 expression while limiting systemic exposure [187]. In parallel, engineered IL‐12 constructs, such as the tumor‐targeting immunocytokine NHS‐IL12, have demonstrated improved safety profiles and encouraging preclinical and early clinical activity, including synergy with ICIs [186].

IL‐18 is a strong immune‐activating cytokine with limited clinical activity when administered to patients due to rapid neutralization through the decoy receptor IL‐18BP and a short half‐life [188]. New engineered forms—such as decoy‐resistant variants (e.g., DR‐18) and Fc‐fused IL‐18 molecules—overcome these barriers by maintaining activity despite high IL‐18BP levels and improving pharmacokinetics. These next‐generation IL‐18 agents demonstrate potent antitumor effects in preclinical models, including enhanced CD8^+^ T‐cell and NK‐cell function, reduced exhaustion, and activity even in checkpoint‐refractory tumors [188]. In RCC models, DR‐18 synergized with anti‐CTLA‐4 (but not anti–PD‐1), supporting further clinical evaluation of this combination [189].

IL‐21 acts by stimulating cytotoxic T lymphocytes and natural killer cells, enhancing antibody production, and modulating the tumor microenvironment through the JAK/STAT pathway [190]. Unlike other members of the common γ chain family, IL‐21 does not expand regulatory T (Treg) cells by suppressing Foxp3 expression, resulting in lower levels of lethal toxicity and systemic side effects [191]. Monotherapy of IL‐21 did show some clinical benefit in first‐line metastatic melanoma patients [192], but due to its preferable safety profile, many alterations and combinations are being tested [190, 193, 194].

In conclusion, cytokine‐based therapies are emerging as important immune activators, fueled by technological advances that address the limitations of native cytokines. Recent developments enable more selective activation of cytotoxic lymphocytes with reduced toxicity and diminished Treg expansion. These next‐generation agents not only broaden the therapeutic window but also show encouraging synergy with ICIs, including in settings of prior treatment resistance.

### 5.2 Cancer Vaccines

Effective cancer vaccines work by enhancing antigen presentation to expand T‐cells specific to target antigens. When tumor antigens are presented by activated antigen‐presenting cells (APCs), they drive massive proliferation of antigen‐specific T‐cells, enabling them to locate and eliminate tumor cells expressing those antigens. In response, tumors develop resistance mechanisms—such as altering antigen expression, preventing T‐cell infiltration into the tumor, or upregulating immune checkpoints (e.g., PD‐L1)—which collectively function to suppress the attacking T‐cells.

**FIGURE 8:**
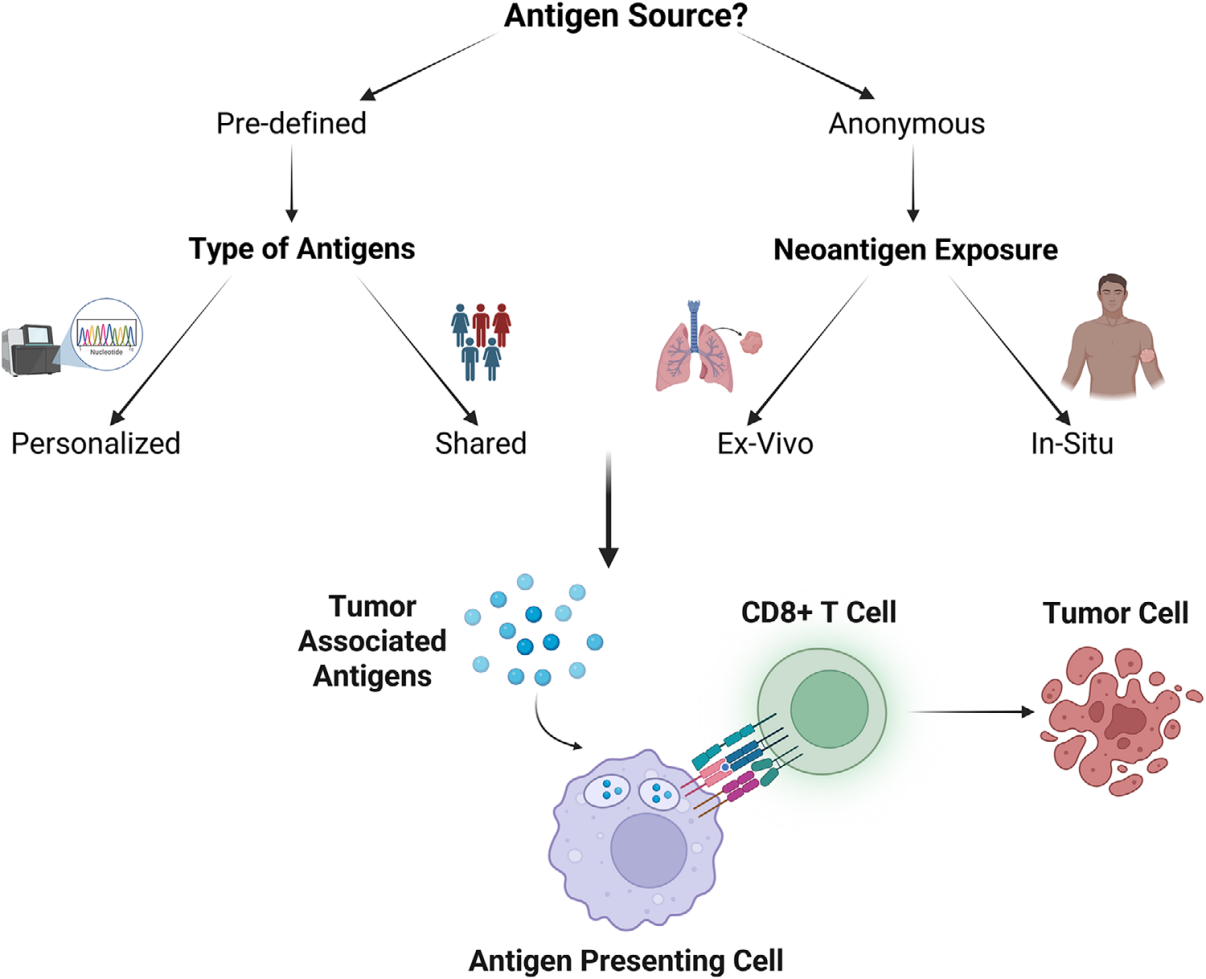
Different Types of Cancer Vaccines Cancer vaccines can be classified based on antigen source and mode of neoantigen exposure. Vaccines may target predefined antigens, including personalized or shared tumor‐associated antigens, or rely on anonymous antigen exposure through ex vivo or in situ approaches. These strategies aim to enhance antigen presentation by antigen‐presenting cells and promote tumor‐specific CD8^+^ T‐cell activation and antitumor immune responses.

Targetable antigens may consist of tumor‐specific neoantigens, which arise from mutated proteins within cancer cells, or tumor‐associated antigens, which originate from normal proteins but are overexpressed in tumors relative to healthy tissues (Figure 8). A central challenge in cancer vaccine development is identifying antigens that generate strong antitumor immune responses while avoiding self‐reactivity and the risk of autoimmune toxicity.

In this context, personalized neoantigen vaccines are particularly attractive because they can induce de novo T‐cell responses against patient‐specific tumor neoantigens while largely avoiding the constraints of central tolerance that often limit responses to shared self‐antigens [195].

Emerging clinical data suggest that personalized neoantigen vaccines may have broader relevance across biologically diverse solid tumors. In multiple disease settings, these individualized approaches have been shown to induce neoantigen‐specific T‐cell responses, in some cases with evidence of immunologic durability and early signals of clinical benefit.

The most clinically advanced example to date is intismeran autogene (formerly mRNA‐4157/V940), an individualized mRNA vaccine encoding up to 34 patient‐specific neoantigens and evaluated with pembrolizumab in the phase 2b KEYNOTE‐942 trial in high‐risk, completely resected melanoma [196]. This approach was associated with durable improvement in recurrence‐free and distant metastasis‐free survival compared with pembrolizumab alone at 3‐years of follow‐up, supporting its continued evaluation in an ongoing phase 3 trial [197]. These findings should nevertheless be interpreted with caution, given the trial's design limitations, including its modest size, one‐sided statistical design, pandemic‐related randomization constraints, and greater censoring in the vaccine arm.

Autogene cevumeran represents another individualized mRNA‐based approach and has shown encouraging activity in the adjuvant setting in pancreatic adenocarcinoma, tested with atezolizumab and standard of care mFOLFIRINOX chemotherapy [198]. In the phase 1 study, vaccine‐induced neoantigen‐specific T‐cell responses were observed in 50% of treated patients. More recent follow‐up has also suggested favorable long‐term survival among immune responders, although these updated data remain preliminary and have not yet been fully reported in the peer‐reviewed literature. Based on these encouraging data, a randomized, multicenter phase 2 trial is currently enrolling patients, and Autogene cevumeran is now also being investigated in early‐phase trials across additional solid tumors, including melanoma and bladder cancer.

Additional support for this approach comes from early studies in triple‐negative breast cancer and clear‐cell renal cell carcinoma [199, 200]. Despite substantial differences in tumor biology and mutational landscape, both studies showed that individualized vaccines can induce durable, mutation‐directed T‐cell responses. In TNBC, responses were largely de novo, targeted multiple neoantigens, and remained detectable after long‐term follow‐up, whereas in RCC, vaccine‐induced immunity was observed in all treated patients and included responses against canonical driver alterations such as VHL, PBRM1, BAP1, KDM5C, and PIK3CA. Although these studies were small and nonrandomized, together they suggest that personalized vaccination may have broader relevance across solid tumors.

The rationale for combining personalized vaccines with ICIs lies in their complementary effects on distinct steps in the tumor‐immune interaction. Whereas vaccination can generate or expand neoantigen‐specific T‐cell populations, particularly in immunologically “cold” tumors with limited endogenous priming, checkpoint blockade may preserve the function of these newly generated cells once they encounter immunosuppressive mechanisms within the tumor microenvironment. ICIs may also counteract the adaptive upregulation of inhibitory pathways that can accompany vaccine‐driven immune activation. Conversely, in the absence of effective priming, ICIs may have little tumor‐reactive T‐cell substrate to reactivate, providing a strong mechanistic basis for combined vaccine‐checkpoint blockade strategies [201].

Despite these encouraging advances, several barriers still limit the transition of personalized neoantigen vaccines from highly innovative clinical trials to routine oncology practice. Key challenges include the uncertainty of neoantigen prediction and selection, individualized manufacturing timelines and scalability, unresolved questions regarding optimal dose, schedule, route, and sequencing with combination therapies, and the regulatory and cost challenges created by an individualized production process [202].

An alternative vaccination approach is anonymous‐antigen vaccines, in which vaccines cause the tumors to release their antigens to promote antigen presentation and T‐cell activation and expansion [203]. With this strategy, the targeted antigens might remain unknown or anonymous, and these vaccines are not dependent on antigen identification and personalized vaccine preparation. Another advantage of anonymous‐antigen vaccines is the potential to present an array of antigens broader than what omics technologies and computational methods can detect. Examples of anonymous‐antigen vaccines administered to humans include vaccines against heat shock proteins (NCT03018288), studies of pulsed dendritic cells, transduction of tumor cells ex vivo to overexpress GM‐CSF, and studies using TLR agonists, as reviewed [204].

Because personalized vaccines require substantial time and resources that may not be feasible for all patients or institutions, efforts to develop vaccines targeting shared antigens are also being pursued. An emerging related strategy is the development of off‐the‐shelf vaccines against recurrent public neoantigens, particularly oncogenic driver mutations such as KRAS. In the phase 1 AMPLIFY‐201 trial, the ELI‐002 2P vaccine, targeting KRAS G12D and G12R, induced mKRAS‐specific T‐cell responses in 84% of patients with minimal residual pancreatic or colorectal cancer, with responses associated with improved relapse‐free and overall survival. Although these data remain early and nonrandomized, they support the potential of shared neoantigen vaccines as a practical bridge between fully personalized vaccines and conventional shared‐antigen approaches [205].

Historically, shared‐antigen vaccine strategies have primarily focused on tumor‐associated antigens rather than recurrent driver neoantigens. Sipuleucel‐T was the first cancer vaccine approved by the FDA. It is a shared antigen vaccine containing prostatic acid phosphatase (PAP), which is more highly expressed in prostate cancer cells compared with normal prostate tissue, and is fused to GM‐CSF and cocultured with patient‐derived dendritic cells. It demonstrated improved survival compared with placebo in the IMPACT trial [206], but its clinical relevance today is limited due to advances in anti‐androgen therapies.

OSE2101 is a therapeutic multiantigen vaccine designed to induce cytotoxic T lymphocytes against five tumor‐associated antigens—HER‐2/neu, CEA, MAGE‐2, MAGE‐3, and p53—that are frequently overexpressed in non‐small cell lung cancer and presented in patients with the HLA‐A2 phenotype [207]. The study did not complete accrual due to the COVID‐19 pandemic and was therefore underpowered, but it did show a four‐month survival advantage in a planned subgroup analysis of patients with secondary immune‐checkpoint–blockade resistance (failure after ≥12 weeks of second‐line monotherapy).

Cancer vaccinations are also being developed against immunosuppressive molecules, to alter the tumor microenvironment and improve the response to other immunomodulatory treatments. IO102/IO103 is a vaccine against indoleamine 2,3‐dioxygenase (IDO) and PD‐L1. IDO is a rate‐limiting enzyme that promotes immunosuppressive effects by regulating the consumption of tryptophan and the accumulation of kynurenine in the tumor microenvironment [208]. In melanoma, the vaccine had an 80% response rate in the anti‐PD1 therapy‐naive cohort, with mPFS of 25.5 months and mOS not reached at data cut‐off [209]. The treatment appeared to also be effective in patients with unfavorable characteristics, such as PDL‐1 negative, high LDH and M1C disease. Unfortunately, the treatment did not demonstrate clinical benefit in patients after progression on anti‐PD1 therapy [210].

Anonymous‐antigen vaccines are divided to two main groups – ex vivo‐based on a tumor biopsy, or in situ, as in oncolytic viruses, including intra‐tumoral T‐VEC [211] that was FDA approved for melanoma patients. In situ vaccines are limited by the need for accessible tumors and challenges in overcoming the immunosuppressive microenvironment, and their utility has therefore been limited to date.

## 6 Challenges and Future Directions in Cancer Immunotherapy

Over the past decade, cancer immunotherapy has transformed the therapeutic landscape across multiple solid tumors. This review highlights the expanding repertoire of immune‐based treatments—including checkpoint inhibitors, antibody‐drug conjugates, cellular therapies, and cancer vaccines—as well as the biological principles that underlie their mechanisms of action and clinical efficacy. Despite these major advances, substantial challenges remain. Many patients do not derive durable benefit, resistance often emerges, and the integration of novel immunotherapy combinations into increasingly earlier stages of disease raises new questions regarding optimal sequencing, patient selection, trial design, and long‐term outcomes.

### 6.1 Variability in Resistance Mechanisms

One of the most pressing challenges is the heterogeneity in resistance to immune checkpoint inhibition. The Society for Immunotherapy of Cancer has convened consensus panels to broadly define categories of resistance to anti‐PD‐1 monotherapy, and combinations with other immunotherapies, targeted therapies or chemotherapy [212, 213, 214, 215, 216]. Although these definitions still require empirical validation, their intention is to harmonize trial design and improve patient selection. Other efforts are ongoing to define resistance by biological mechanisms and biomarkers.

Primary resistance, defined as progression of disease within 12 weeks, may arise from tumor‐intrinsic factors or the presence of a profoundly immunosuppressive tumor microenvironment (TME) enriched with myeloid‐derived suppressor cells and regulatory T‐cells. Conversely, secondary resistance may involve T‐cell exhaustion or adaptive changes in tumor metabolism. Adaptive resistance has been proposed as a process in which the cancer is recognized by the immune system but protects itself by adapting to the immune attack, and may be characterized by an initial radiologic response or mixed response [217]. A subset of patients has a late response, sometimes after initial progression. Differentiation between the possibility of a late response versus actual progression is difficult and must be correlated with the clinical status of the patient.

Distinguishing between primary and secondary resistance is essential for guiding subsequent management. While patients with secondary resistance may benefit from escalation of current treatment (such as switching to dual immune checkpoint blockade) [218], or by adding other methods to the main systemic treatment such as targeted or local radiotherapy [219, 220], those with primary resistance may require a complete change in course of treatment—such as tumor‐targeting approaches or employing adoptive cell therapies [220, 221, 222]. To date, we do not have enough individualized biomarkers to predict response to immunotherapy. While PD‐L1, TMB, and MSI/dMMR are routinely used to predict response, their absence does not always correlate with the potential of response to immunotherapy, especially in tumors that are otherwise highly immunogenic. Other developing directions are related to characterizing the tumor microenvironment (including the presence of tumor‐infiltrating lymphocytes and tertiary lymphoid structures), identification of neoantigens, epigenetic signatures, and genomic alterations [223]. Circulating tumor DNA technologies sensitivity is improving and might be helpful for identifying cases of pseudoprogression and early identification of secondary resistance or early disease progression in the adjuvant setting [224].

### 6.2 Sequencing of Treatments After Early Use of Immunotherapy in Localized Disease

As immunotherapy becomes standard in the neoadjuvant and adjuvant settings, we are now seeing an emerging population of patients who are experiencing metastatic recurrences after prior exposure to ICIs. From a biological perspective, additional work is needed to determine mechanisms of resistance in these patients, which might be quite different from patients with metastatic disease who develop resistance on or off therapy.

In the metastatic setting in diseases such as melanoma, where multiple immune therapies are approved, it remains unclear whether an aggressive approach upfront is superior to salvage therapy after less toxic frontline regimens in terms of long‐term survival, physical and financial toxicity.

Emerging studies of mechanisms of resistance will likely inform future approaches. An Australian study that followed the clinical course of 103 melanoma patients treated with adjuvant anti‐PD‐1 reported a 36% recurrence rate within less than 20 months [225]. Studies of the primary tumor revealed that bystander T‐cells (CD39‐CD103‐PD‐1‐CD8+) comprised a significantly greater proportion of T‐cells in patients who developed recurrence, compared with CD39+ tumor‐resident memory cells (CD39+CD103+PD‐1+CD8+) that comprised a significantly higher proportion of CD8+ T‐cells in recurrence‐free patients. Further validation is needed to determine if this population of T‐cells can represent a biomarker of recurrence‐free survival or an indicator of the need for therapeutic manipulation for CD39‐ T‐cell‐enriched tumors, either in the adjuvant setting or later in metastatic disease.

Overall, our understanding of the biological drivers of recurrence after prior immunotherapy exposure remains limited. As the use of neoadjuvant and adjuvant ICIs expands, there will be a need to redefine subsets of frontline metastatic patients and stratify them better in clinical trials based on the biology of their tumors. More patients with intrinsic or early resistance will be revealed earlier in the disease course.

### 6.3 De‐Escalation Strategies for Immune Checkpoint Inhibitors

Initial development of ICIs was geared toward identifying the maximum benefit at the recommended phase 2 dose of a regimen, and long‐term effects were not sufficiently considered. Most pivotal trials have included treatment until progression, toxicity, or a maximum of 2 years [226]. However, shortening the course of treatment for patients who achieve a confirmed response has been shown to be safe in patients with metastatic melanoma after at least 6 months of therapy, with a low incidence of relapse after a median follow‐up of approximately 2 years from discontinuation [227]. A real‐world study based on the EUMelaReg registry including 1291 patients, showed PFS and OS benefit for patients continuing treatment for >12 months or until progression after achieving PR or CR compared to patients treated for <6 months, with a trend of benefit also in the 6–12 months treatment group [204]. However, these studies are inherently limited, and prospective trials are needed when feasible. Importantly, one cannot extrapolate from melanoma to tumor types that are not associated with a potential cure from immunotherapy. In potentially curable diseases such as melanoma, indefinite immunotherapy exposes patients to unnecessary costs and healthcare burden, but more importantly, toxicity that might be chronic or fatal.

De‐intensifying the cumulative dose of anti‐CTLA‐4 has been done in melanoma. Ipilimumab containing combinations are far more toxic than monotherapy or anti‐PD‐1 and LAG‐3 combinations [18]. CheckMate 511 compared two dosing regimens, nivolumab 1mg/kg with ipilimumab 3mg/kg as standard of care versus the inverse ratio [19]. The primary endpoint was the incidence of high‐grade toxicity, which was significantly lower with the lower dose of ipilimumab, as were discontinuation rates. Descriptive analyses, even if not powered to address this question, revealed that both groups demonstrated high 3‐y OS rates that were numerically similar, but long‐term OS data have not been published.

Another approach to reduce toxicity was tested in the ADAPT‐IT trial, a relatively small phase 2 trial, which investigated early discontinuation of ipilimumab (at 3 mg/kg) after two cycles in patients with early radiographic response [228]. Patients with evidence of a favorable antitumor effect (FATE; defined as growth of target lesions by ≤4% and the absence of new lesions) transitioned to maintenance nivolumab alone, and those who did not were assigned to complete two additional treatments with ipilimumab. The study included 70 patients, 23% of whom had mucosal melanoma, and 13% had received prior systemic therapy (mainly adjuvant), suggesting a more aggressive disease profile. Disease outcomes, including ORR and duration of response, were similar to the results described in CheckMate 067, but unfortunately, the rates of grade 3–5 toxicity were similar among patients with and without FATE. This suggests that the initial dose intensity at the beginning of treatment may be the reason for severe toxicity, rather than the treatment frequency. These findings warrant validation in a larger, multi‐institutional trial, and biomarker driven approaches, such as de‐escalation based on circulating DNA and novel imaging approaches, might further our ability to de‐escalate therapy.

### 6.4 Conversion of Cold to Hot Tumors

Hot tumors—also referred to as immune‐inflamed tumors—exhibit high immunogenicity and demonstrate responsiveness to immunotherapy. They contain abundant cytotoxic T‐cell infiltration, tertiary lymphoid structures, and a broadly pro‐inflammatory environment, all of which promote effective antitumor immunity and enhance clinical outcomes with ICIs [229].

Cold tumors lack intrinsic immunogenicity and are therefore unresponsive or minimally responsive to immunotherapy, including ICIs. These tumors are marked by poor immune cell infiltration and a profoundly immunosuppressive TME that supports immune evasion and resistance to ICIs. Key biological features include low tumor mutational burden, dense stromal barriers, impaired antigen presentation, and abnormal vasculature. Cold tumors are further categorized to immune‐desert tumors, with an absence of T‐cells in both the core and invasive margin; altered‐excluded tumors, in which T‐cells are confined to stromal boundaries due to physical barriers; and altered‐immunosuppressed tumors, defined by low T‐cell density with functional inhibition [229]. A key distinction between hot and cold states is the burden of immune suppressive cells, which are enriched in cold tumors and contribute to reduced immunogenicity(231).

As most cold tumors do not respond adequately to immune checkpoint blockade, even at treatment initiation, newer approaches are needed. A full discussion of advanced treatment strategies is beyond the scope of this review, but includes strategies such as low‐dose radiation and chemotherapy to enhance antigen spread. HLA‐deficient tumors that present a colder microenvironment at baseline, may benefit from a combination of chemotherapy and immunotherapy to induce a strong immune response involving tertiary lymphoid structures [144]. Integrating cutting‐edge immunotherapeutic approaches will likely involve sophisticated technologies and combination strategies, including—but not limited to—epigenetic reprogramming, CAR‐T‐cell therapy, immunoliposomes, and vaccine‐based interventions [229, 230, 231, 232].

### 6.5 Clinical Trial Considerations

Clinical trials, industrial and investigator‐initiated, are an inseparable part of advancements in immunotherapy. Immunotherapy trials differ from those of standard chemotherapy in many aspects, including concurrent use of corticosteroids, underlying autoimmunity, phase I trial designs in which long‐term toxicity might not be reflected during the dose‐limiting toxicity evaluation period, and radiographic response criteria.

Many pivotal clinical trials in oncology, including immunotherapy, lack crossover opportunities. This is due to a range of reasons—from feasibility of access to therapy in different countries to avoiding confounding factors for survival analysis. This needs to be weighed against potential benefits to trial participants. Analysis of 23 first‐line clinical trials in NSCLC revealed that even though 15 of them started after the approval of second‐line ICI therapy, only 40.8% of patients in the control arms received ICI after progression of disease [233]. In‐trial crossover is a means to ensure that all participants have access to the best standard‐of‐care, although it increases trial costs. In‐trial crossover can also be informative. For example, EORTC1325/KEYNOTE‐054 [91] assessed adjuvant pembrolizumab versus placebo in patients with high‐risk melanoma. Patients from the placebo arm could crossover to receive pembrolizumab upon progression, and 50% of progressing patients crossed over. Patients from the therapeutic arm could be rechallenged with pembrolizumab. Importantly, while RFS and DMFS were superior on the pembrolizumab arm, OS at 7 years was identical, although the number of deaths was low. This important finding is supported by the results of SWOG S1404 in which survival was identical in patients receiving pembrolizumab or standard of care ipilimumab or interferon [92].

While improved survival is the primary goal of adjuvant therapy, almost all adjuvant immunotherapy trials did not demonstrate OS benefit [234, 235], and with emerging salvage therapies in the metastatic setting, this might never happen, especially in intermediate‐risk disease. Therefore, decisions regarding adjuvant treatment must be individualized, taking into consideration the features of the disease, risk factors of the patient himself, and possible treatment lines if recurrence happens. Due to the possible long‐term toxicity of immune checkpoint inhibitors, other adjuvant regimens void of long‐term toxicities should be considered [236].

KEYNOTE‐564 included patients with high‐risk clear cell RCC and concluded that adjuvant pembrolizumab is associated with a significant improvement in OS, as compared with placebo, although follow‐up was short and the number of events was very small [237]. Moreover, there were some concerns about informative censoring, as early and disproportionate loss to follow‐up in the pembrolizumab arm may have affected results. When this imbalance was addressed using a modified time‐to‐treatment‐failure approach—treating excess censoring as an event—the observed benefit was no longer statistically significant. In addition, suboptimal use of immunotherapy in relapsing control‐arm patients and lack of consistency with other negative adjuvant immunotherapy trials further question the validity of survival data [238]. Censoring imbalance analysis was further tested in several phase 3 trials of first‐line ICIs in advanced urothelial carcinoma, and the authors concluded that differential censoring could explain the inconsistent benefit reported in the evaluated trials [239]. To avoid informative censoring, future trials should ensure balanced and complete follow‐up across study arms, specify OS or an adequate surrogate as its primary endpoint, and guarantee optimal post‐recurrence therapy for all control‐arm patients [238].

Dose intensity for drugs such as ipilimumab is important for enhancing response and survival, where higher doses of 10 mg/kg every 3 weeks are associated with improved outcomes compared to 3 mg/kg [240]. Patients with NSCLC are treated at 1mg/kg every six weeks, and other regimens might use 1mg/kg every 3 weeks. Although higher doses are associated with greater toxicity, study designs should incorporate different doses to assess the effects on long‐term survival in different tumors other than melanoma.

In conclusion, immunotherapy has reshaped the management of solid tumors, yet the field now faces enhanced complexity as its use expands into earlier disease settings and increasingly complex clinical scenarios of resistance. Durable benefit remains limited to a subset of patients, and emerging patterns of primary and secondary resistance highlight the need for deeper biological understanding, refined biomarkers to guide treatment selection, and more sophisticated, next‐generation immunotherapeutic approaches. As neoadjuvant and adjuvant strategies become standard, questions regarding optimal sequencing, management of post‐immunotherapy recurrences, and the safe de‐escalation of therapy are becoming central to clinical decision‐making. At the same time, overcoming the barriers that define cold tumors and designing trials that better reflect real‐world populations—while revisiting trial methodologies—will be essential for generating reliable evidence. Together, these challenges outline the next phase of progress in cancer immunotherapy: translating emerging insights into personalized strategies that maximize efficacy, confronting resistance mechanisms, and minimizing treatment‐related toxicity with the ultimate goal of increasing overall survival.

## Author Contributions

**Shira Gabizon ‐ Peretz**: conceptualization, investigation, writing – original draft, and visualization. **Harriet M. Kluger**: conceptualization, writing – review & editing, supervision and project administration.

## Conflicts of Interest

The authors declare no conflicts of interest.

## Data Availability

Data sharing is not applicable to this article, as no new datasets were generated or analyzed during the current study. The data summarized in the tables and discussed in the manuscript were derived from previously published studies and publicly available sources, as cited in the manuscript.
